# Admission Phenotype Clarifies the Apparent Prognostic Contrast Between TOAST Etiologic Subtypes in Large‐Core Anterior‐Circulation Large‐Vessel Occlusion

**DOI:** 10.1002/cns.71029

**Published:** 2026-07-13

**Authors:** Chengsong Yue, Shihai Yang, Xiongxiong Hou, Jingfu Ma, Xu Xu, Xiaolei Shi, Linyu Li, Jie Yang, Changwei Guo, Jiaxing Song, Wenjie Zi, Rongzong Li

**Affiliations:** ^1^ Department of Neurology No. 924 Hospital of Joint Logistic Support Force of Chinese PLA GuangXi China; ^2^ Department of Neurology Xinqiao Hospital and The Second Affiliated Hospital, Army Medical University (Third Military Medical University) Chongqing China; ^3^ Department of Neurology The Second Hospital & Clinical Medical School, Lanzhou University Lanzhou China

**Keywords:** collateral circulation, endovascular thrombectomy, large‐core ischemic stroke, stroke etiology

## Abstract

**Objective:**

In large‐core anterior‐circulation large‐vessel occlusion (LVO), differing admission clinical‐imaging profiles complicate interpretation of prognostic differences between large‐artery atherosclerosis (LAA) and cardioembolism (CE). We assessed whether these differences persisted after adjustment and whether endovascular thrombectomy (EVT)–outcome associations differed by etiology.

**Methods:**

We analyzed 631 patients with Alberta Stroke Program Early CT Score (ASPECTS) ≤ 5 from the 38‐center MAGIC registry; 404 underwent EVT plus medical therapy and 227 received medical therapy alone. The primary outcome was 90‐day modified Rankin Scale (mRS) 0–3. Mixed‐effects models assessed etiology–outcome associations and treatment‐by‐etiology interaction. Among EVT‐treated patients, Shapley decomposition quantified domain contributions to attenuation of the LAA–CE contrast.

**Results:**

Among EVT‐treated patients, higher crude odds of mRS 0–3 with LAA (OR 1.59, 95% CI 1.05–2.42) were substantially attenuated after adjustment (aOR 0.92, 95% CI 0.53–1.59). No adjusted LAA–CE contrast was evident with medical therapy alone (aOR 1.12, 95% CI 0.47–2.65). In a separate fixed‐effects decomposition model, the absolute log‐odds contrast was attenuated by 96.2% after inclusion of measured pretreatment domains, with imaging/collateral features, demographics, and stroke severity contributing most. For the secondary mRS 0–2 outcome, adjusted odds favored CE (aOR 0.46, 95% CI 0.25–0.84). No treatment‐by‐etiology interaction was detected (*p* = 0.944).

**Interpretation:**

Admission clinical‐imaging phenotype clarified the apparent LAA–CE prognostic contrast and may help reconcile discordant findings across EVT cohorts. These findings reinforce established clinical‐imaging criteria as the basis for hyperacute EVT selection rather than LAA versus CE etiology alone.

**Trial Registration:**

Chinese Clinical Trial Registry (ChiCTR.org.cn); ChiCTR2100051664; https://www.chictr.org.cn/

## Graphical abstract

In large‐core anterior‐circulation LVO, the crude LAA–CE prognostic contrast was reduced by 96.2% after adjustment for measured admission phenotype in a model‐based decomposition. Adjusted EVT–outcome associations were similar across subtypes, arguing against modifying EVT selection solely by LAA versus CE etiology.

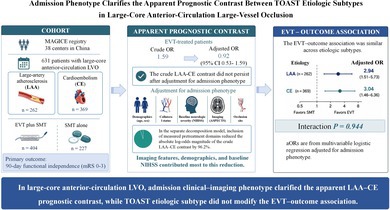

## 1 Introduction

Acute ischemic stroke with a large infarct core—often operationalized as an Alberta Stroke Program Early CT Score (ASPECTS) [1] ≤ 5—due to anterior‐circulation large‐vessel occlusion (LVO) carries mortality rates of 53%–78% under medical management alone [2, 3]. Although recent randomized trials established the benefit of endovascular thrombectomy (EVT) in selected patients with large‐core stroke [4, 5, 6, 7], outcomes remain heterogeneous [8]. This variability highlights the need to identify which information available at presentation is most relevant to prognosis and hyperacute decision‐making. Neurologic severity and admission imaging features—including core burden, collateral status, and occlusion characteristics—are established determinants of outcome [9, 10, 11]. The prognostic role of stroke etiology, particularly large‐artery atherosclerosis (LAA) versus cardioembolism (CE), remains less clear [12, 13, 14].

Previous studies of etiologic subtypes defined by the Trial of ORG 10172 in Acute Stroke Treatment (TOAST) classification [15] were conducted in anterior‐circulation EVT cohorts not specifically focused on large‐core stroke, and their findings were inconsistent. The SITS registry reported worse adjusted outcomes in LAA than in CE [12], whereas other multicenter cohorts found broadly similar 90‐day outcomes [16]. This discordance leaves the clinical relevance of LAA versus CE in the hyperacute setting uncertain. Interpretation is further complicated because the two subtypes differ in demographic profile, collateral recruitment, baseline neurologic severity, and occlusion characteristics [17, 18]. However, in large‐core stroke, the extent to which the crude LAA–CE contrast changes after accounting for clinical‐imaging phenotype, and the domains contributing most to that change, remain poorly defined. This gap is particularly important in large‐core stroke, where prognosis is strongly shaped by infarct burden, neurologic severity, collateral status, and occlusion pattern, while pivotal EVT trials established eligibility primarily through clinical and imaging criteria rather than TOAST subtype [4, 5, 6, 7]. Whether etiology retains clinically relevant prognostic information beyond admission phenotype and whether the association between EVT and outcome differs by etiology therefore remain unresolved.

In this multicenter cohort of patients with large‐core anterior‐circulation LVO, we assessed whether the apparent LAA–CE prognostic contrast persisted after accounting for admission clinical‐imaging phenotype. We further quantified the pretreatment domains contributing to the crude‐to‐adjusted change in this contrast and tested whether the association between EVT and outcome differed by etiology.

## 2 Methods

### 2.1 Study Design and Patient Population

This study was a secondary analysis of the MAGIC registry (ChiCTR2100051664), a prospective, multicenter, nationwide observational cohort involving 38 stroke centers in China [19]. The registry consecutively enrolled patients with acute ischemic stroke due to large‐vessel occlusion presenting within 24 h of symptom onset. For the present analysis, we included patients treated between November 1, 2021, and February 8, 2023, with anterior‐circulation occlusion involving the intracranial internal carotid artery or the M1/M2 segment of the middle cerebral artery. Symptom onset was defined as witnessed onset or last known well. The registry protocol was approved by the ethics committee of Xinqiao Hospital, Army Medical University, and by the institutional review board at each participating center. Written informed consent was obtained from each patient or their legally authorized representative.

Eligible patients were adults aged ≥ 18 years with a large infarct core, defined as an ASPECTS ≤ 5 on baseline noncontrast CT, and stroke etiology classified as large‐artery atherosclerosis (LAA) or cardioembolism (CE) according to the TOAST criteria [15]. We excluded patients with a prestroke modified Rankin Scale [20] (mRS) score > 2, unavailable 90‐day outcome assessment, or etiologies other than LAA or CE, including undetermined and other determined etiologies.

**FIGURE 1:**
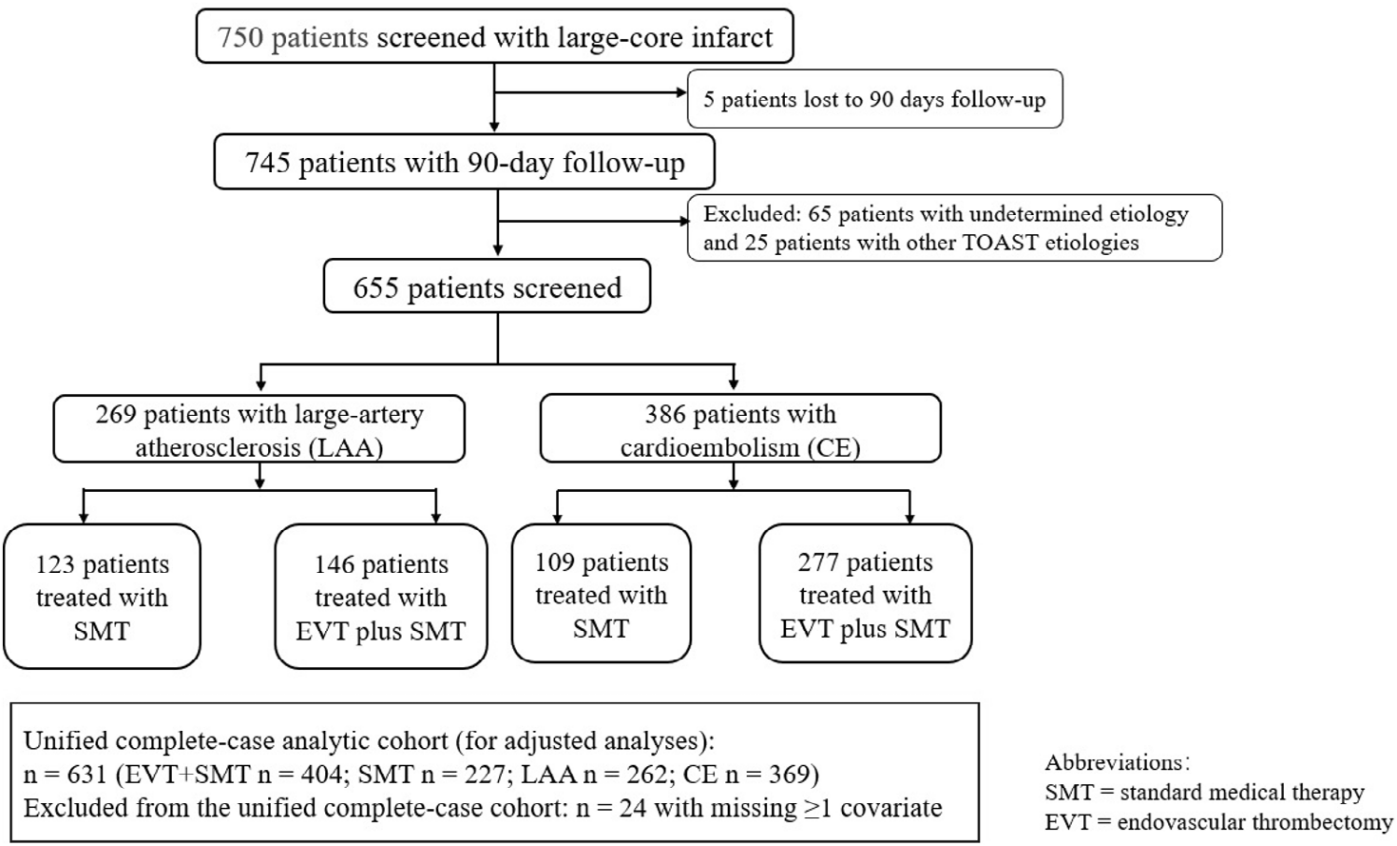
Study flow diagram and analytic cohorts. Of 750 screened patients, 5 without 90‐day follow‐up were excluded. Among the remaining 745 patients, 90 with etiologies other than large‐artery atherosclerosis or cardioembolism were excluded (undetermined etiology, *n* = 65; other determined etiologies, *n* = 25), leaving 655 eligible patients. Of these, 423 received endovascular thrombectomy plus standard medical therapy and 232 received standard medical therapy alone. After excluding 24 patients with missing data for at least 1 prespecified covariate required for adjusted analyses, the unified complete‐case cohort included 631 patients (endovascular thrombectomy, *n* = 404; standard medical therapy alone, *n* = 227). CE, cardioembolism; EVT, endovascular thrombectomy; LAA, large‐artery atherosclerosis; LVO, large‐vessel occlusion; SMT, standard medical therapy; TOAST, Trial of ORG 10172 in Acute Stroke Treatment.

Treatment strategy—endovascular thrombectomy (EVT) plus standard medical therapy (SMT) or SMT alone—was determined by the treating team during routine clinical care according to patient‐specific clinical and imaging features and local practice. Cohort derivation is shown in Figure 1. Additional details on eligibility criteria, treatment classification, and the complete‐case analytic dataset are provided in Method S1.

### 2.2 Etiological Classification

Stroke etiology was classified using TOAST‐based categories on the basis of all available clinical, vascular, and cardiac diagnostic information [15]. LAA was assigned when > 50% atherosclerotic stenosis or atherosclerotic occlusion of a relevant extracranial or intracranial artery supplying the infarct territory was judged to be the most likely cause of the index stroke. CE was assigned when a recognized high‐risk cardiac source of embolism was judged to be the most likely cause after consideration of competing large‐artery sources.

Etiologic subtype was independently assigned by two trained stroke neurologists using all available diagnostic information, with disagreements resolved by a third senior neurologist. Patients classified as having undetermined or other etiologies were excluded from the present analysis.

### 2.3 Data Collection and Definitions

Pretreatment variables included demographics, vascular risk factors and medical history, admission laboratory measures, intravenous thrombolysis status, stroke severity assessed using the National Institutes of Health Stroke Scale (NIHSS) [21], infarct extent assessed using ASPECTS on baseline noncontrast CT [1], and occlusion site on baseline vascular imaging. Baseline characteristics are summarized in Table 1. Detailed variable and outcome definitions are provided in Method S2.

**TABLE 1:** Baseline characteristics by etiology within treatment strata in the complete‐case cohort.

	EVT cohort			SMD^a^	Total (*n* = 227)	SMT‐only cohort		SMD^a^	SMD^b^
	Total (*n* = 404)	LAA stratum (*n* = 143)	CE stratum (*n* = 261)			LAA stratum (*n* = 119)	CE stratum (*n* = 108)		
Demographics									
Age, years, median (IQR)	70 (61, 78)	67 (58, 76)	72 (64, 79)	0.290	72 (65, 79)	70 (62, 76.5)	75 (68, 80.3)	0.468	0.167
Sex, female, *n* (%)	179 (44.3)	45 (31.5)	134 (51.3)	0.403	107 (47.1)	48 (40.3)	59 (54.6)	0.286	0.057
Laboratory and physiological measures									
Glucose, mmol/L, median (IQR)	7.2 (6.0, 9.0)	7.3 (6.0, 9.4)	7.2 (6.1, 8.6)	0.101	7.1 (6.0, 8.6)	6.8 (5.7, 9.2)	7.2 (6.5, 8.2)	0.236	0.007
Admission blood pressure, mmHg, median (IQR)									
Systolic	146.0 (128.0, 166.0)	150.0 (133.0, 169.5)	143.0 (126.0, 161.0)	0.246	156.0 (136.5, 179.0)	154.0 (135.5, 181.5)	156.0 (137.0, 176.3)	0.056	0.407
Diastolic	86.0 (75.0, 96.0)	88.0 (79.5, 95.5)	84.0 (75.0, 96.0)	0.048	89.0 (80.0, 102.0)	86.0 (80.0, 98.0)	92.0 (80.0, 104.3)	0.213	0.281
Medical history, *n* (%)									
Hypertension	250 (61.9)	105 (73.4)	145 (55.6)	0.373	164 (72.2)	94 (79.0)	70 (64.8)	0.315	0.221
Hyperlipidemia	83 (20.5)	32 (22.4)	51 (19.5)	0.070	32 (14.1)	18 (15.1)	14 (13.0)	0.062	0.170
Diabetes	68 (16.8)	40 (28.0)	28 (10.7)	0.437	49 (21.6)	35 (29.4)	14 (13.0)	0.403	0.121
Smoking	122 (30.2)	57 (39.9)	65 (24.9)	0.320	64 (28.2)	44 (37.0)	20 (18.5)	0.412	0.044
Atrial fibrillation	205 (50.7)	18 (12.6)	187 (71.6)	1.196	101 (44.5)	16 (13.4)	85 (78.7)	1.309	0.125
Stroke characteristics									
ASPECTS, median (IQR)	4.0 (2.0, 5.0)	4.0 (2.5, 5.0)	4.0 (2.0, 5.0)	0.158	3.0 (1.0, 5.0)	4.0 (1.5, 5.0)	2.0 (0.0, 4.0)	0.451	0.389
NIHSS, median (IQR)	17.0 (14.0, 21.0)	16.0 (13.0, 20.0)	18.0 (14.0, 21.0)	0.253	16.0 (13.0, 21.0)	15.0 (12.5, 20.0)	17.0 (14.8, 23.3)	0.367	0.007
Intravenous thrombolysis, *n* (%)	100 (24.8)	42 (29.4)	58 (22.2)	0.163	70 (30.8)	37 (31.1)	33 (30.6)	0.012	0.136
Occlusion site, *n* (%)				0.260				0.395	0.327
internal carotid	163 (40.3)	46 (32.2)	117 (44.8)		58 (25.6)	21 (17.6)	37 (34.3)		
M1 segment	197 (48.8)	79 (55.2)	118 (45.2)		145 (63.9)	86 (72.3)	59 (54.6)		
M2 segment	44 (10.9)	18 (12.6)	26 (10.0)		24 (10.6)	12 (10.1)	12 (11.1)		
ASITN/SIR, *n* (%)				0.566				NA	NA
0–1	194 (48.0)	47 (32.9)	147 (56.3)		NA	NA	NA		
2	138 (34.2)	52 (36.4)	86 (33.0)		NA	NA	NA		
3–4	72 (17.8)	44 (30.8)	28 (10.7)		NA	NA	NA		

*Note:* Data are presented as median (interquartile range) for continuous variables and number (percentage) for categorical variables. For categorical variables with more than 2 levels, standardized mean differences were calculated using the multilevel extension based on differences in category proportions. Standardized mean differences for continuous variables were calculated using the original continuous values. An absolute standardized mean difference > 0.10 was considered indicative of meaningful imbalance. ASITN/SIR collateral grade was assessed angiographically and was therefore available only in EVT‐treated patients.

Abbreviations: ASITN/SIR, American Society of Interventional and Therapeutic Neuroradiology/Society of Interventional Radiology; ASPECTS, Alberta Stroke Program Early CT Score; BP, blood pressure; CE, cardioembolism; EVT, endovascular thrombectomy; IQR, interquartile range; IVT, intravenous thrombolysis; LAA, large‐artery atherosclerosis; NIHSS, National Institutes of Health Stroke Scale; SMD, standardized mean difference; SMT, standard medical therapy.

^a^ Standardized mean difference comparing LAA with CE within the corresponding treatment stratum.

^b^ Standardized mean difference comparing the overall EVT‐treated cohort with the SMT‐only cohort.

In EVT‐treated patients, collateral status was graded on pretreatment angiography using the American Society of Interventional and Therapeutic Neuroradiology/Society of Interventional Radiology (ASITN/SIR) collateral grading scale [22] and categorized as 0–1 (poor), 2 (moderate), or 3–4 (good). EVT workflow and procedural variables, including puncture‐to‐final angiographic assessment time, first‐line thrombectomy technique, and number of device passes, were collected for descriptive purposes and were not included as pretreatment covariates in the primary outcome models.

### 2.4 Imaging Adjudication and Quality Control

Baseline ASPECTS and occlusion site, together with pretreatment angiographic collateral grade in EVT‐treated patients, were centrally adjudicated by an imaging core laboratory blinded to clinical outcomes and etiological classification. Reperfusion status and hemorrhagic transformation on postprocedural or follow‐up imaging were adjudicated using the same core‐laboratory process. Data abstraction was cross‐checked by two investigators, and discrepancies were resolved by consensus with a third investigator.

### 2.5 Outcome Assessment

Clinical outcomes were assessed at 90 days by trained, certified assessors using in‐person visits or structured telephone interviews. Assessors were not involved in acute care and were blinded to baseline etiological classification and imaging data.

The primary outcome was favorable functional outcome, defined as a 90‐day mRS score of 0–3. This threshold was selected to capture clinically meaningful recovery in patients with large‐core stroke and to align with prior large‐core EVT literature, in which mRS 0–2 is less frequently achieved and mRS 0–3 captures avoidance of severe disability in this high‐risk population [23]. Secondary efficacy outcomes included 90‐day mRS 0–2, mRS 0–4, and ordinal shift across the 90‐day mRS distribution (0–6). Safety outcomes were 90‐day all‐cause mortality and symptomatic intracranial hemorrhage (sICH) within 48 h, defined according to the Heidelberg Bleeding Classification [24].

### 2.6 Statistical Analysis

Statistical analyses were performed using R version 4.2.0. All tests were 2‐sided, and effect estimates are reported with 95% confidence intervals (CIs). Secondary outcome and subgroup analyses were considered exploratory, with no adjustment for multiple comparisons. Baseline characteristics were summarized by etiology within each treatment stratum and by treatment group in the overall cohort as median (interquartile range) for continuous variables and number (percentage) for categorical variables. Covariate imbalance was assessed using standardized mean differences (SMDs), with an absolute SMD > 0.10 considered meaningful (Table 1). Procedural and workflow characteristics among EVT‐treated patients are reported in Table S1.

Because missingness in baseline covariates was low, primary analyses used a complete‐case approach. A unified complete‐case cohort required available data on treatment, etiology, treating center, all selected pretreatment covariates, and all analyzed outcomes, thereby maintaining the same participants across endpoints (*n* = 631; EVT, *n* = 404; SMT alone, *n* = 227). Multiple imputation by chained equations with 20 imputed datasets was performed as a sensitivity analysis. Imputation procedures and diagnostic assessments are detailed in Method S3, Figures S1 and S2, and the corresponding estimates are reported in Table S3.

Associations between TOAST etiology and 90‐day outcomes were evaluated separately in the EVT‐treated and SMT‐only strata, with CE as the reference category. Binary outcomes were analyzed using mixed‐effects logistic regression and are reported as odds ratios (ORs); the ordinal mRS distribution was analyzed using mixed‐effects proportional‐odds regression and is reported as a common OR. Models adjusted for age, sex, admission glucose, systolic blood pressure, baseline NIHSS, baseline ASPECTS, intravenous thrombolysis, and occlusion site; angiographic collateral grade was additionally included in the EVT stratum. Treating center was modeled as a random intercept. Because sICH events were sparse, Firth penalized logistic regression was used without a center random effect. Primary results are presented in Table 2, with complete outcome and sensitivity estimates in Tables S2 and S3. Model specifications are provided in Methods S4.1 and S4.2.

**TABLE 2:** Associations between stroke etiology and selected clinical outcomes within treatment strata: Complete‐case analysis.

EVT‐treated cohort (*n* = 404)						
	CE (*n* = 261)	LAA (*n* = 143)	Crude OR (95% CI)	*p* value	Adjusted OR (95% CI)	*p* value
Primary outcome						
mRS 0–3	88 (33.7)	64 (44.8)	1.59 (1.05–2.42)	0.029	0.92 (0.53, 1.59)	0.763
Secondary outcome						
Ordinal mRS shift (0–6)	5 (3, 6)	4 (3, 6)	1.30 (0.90, 1.85)	0.166	0.82 (0.54, 1.25)	0.354
Safety outcome						
Mortality	118 (45.2)	50 (35.0)	0.65 (0.43, 0.99)	0.046	0.99 (0.58, 1.71)	0.98

SMT‐only cohort (*n* = 227)						
	CE (*n* = 108)	LAA (*n* = 119)	Crude OR (95% CI)	*p* value	Adjusted OR (95% CI)	*p* value
Primary outcome						
mRS 0–3	15 (13.9)	29 (24.4)	2.00 (1.00, 3.97)	0.048	1.12 (0.47, 2.65)	0.795
Secondary outcome						
Ordinal mRS shift (0–6)	6 (4, 6)	5 (4, 6)	2.33 (1.41, 3.85)	< 0.001	1.35 (0.77, 2.38)	0.296
Safety outcome						
Mortality	63 (58.3)	44 (37.0)	0.42 (0.25, 0.71)	0.001	0.54 (0.28, 1.02)	0.059

*Note:* Data are presented as number (percentage) for binary outcomes and median (interquartile range) for the ordinal modified Rankin Scale score. Odds ratios (ORs) with 95% confidence intervals (CIs) compare LAA with CE, with CE as the reference category, within each treatment stratum. For binary functional outcomes, an OR greater than 1 indicates higher odds of a favorable outcome with LAA; for mortality, an OR less than 1 indicates lower odds of death with LAA. For the ordinal mRS analysis, a common OR greater than 1 indicates a shift toward lower, more favorable mRS scores with LAA. Adjusted models included age, sex, admission glucose, systolic blood pressure, baseline NIHSS score, baseline ASPECTS, intravenous thrombolysis, and occlusion site; angiographic ASITN/SIR collateral grade was additionally included in the EVT‐treated cohort. Treating center was modeled as a random intercept. Additional outcomes, including 90‐day mRS 0–2, mRS 0–4, and symptomatic intracranial hemorrhage, together with multiple‐imputation analyses, are reported in Tables S2 and S3. Symptomatic intracranial hemorrhage was analyzed using Firth penalized logistic regression because of sparse events.

Abbreviations: ASITN/SIR, American Society of Interventional and Therapeutic Neuroradiology/Society of Interventional Radiology collateral grade; ASPECTS, Alberta Stroke Program Early CT Score; CE, cardioembolism; CI, confidence interval; EVT, endovascular thrombectomy; IQR, interquartile range; LAA, large‐artery atherosclerosis; mRS, modified Rankin Scale; NIHSS, National Institutes of Health Stroke Scale; OR, odds ratio; SMT, standard medical therapy.

**FIGURE 2:**
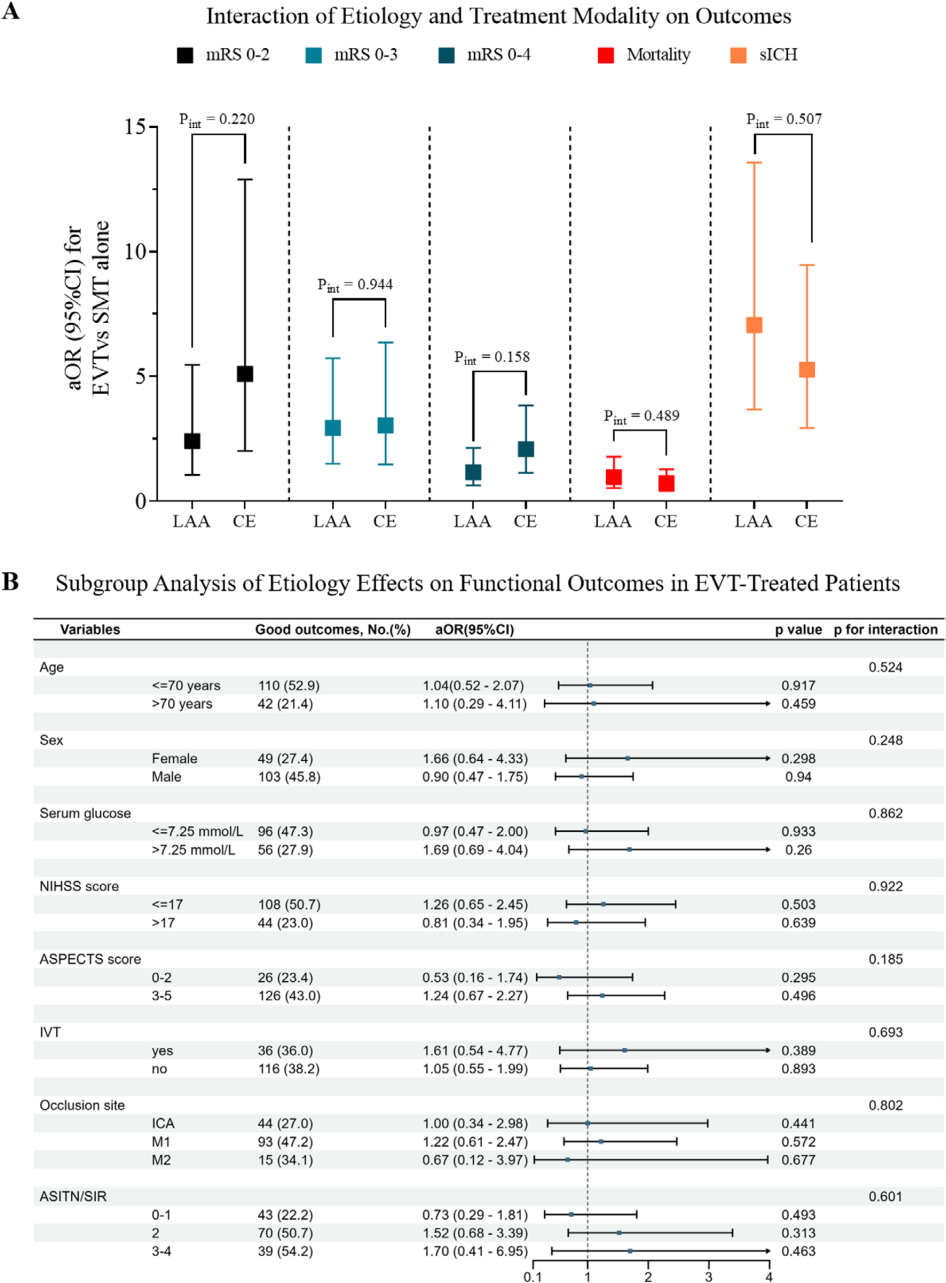
Etiology‐by‐treatment interaction and subgroup analyses. (A) Adjusted odds ratios (ORs) with 95% confidence intervals (CIs) for endovascular thrombectomy (EVT) plus standard medical therapy (SMT) versus SMT alone within the large‐artery atherosclerosis (LAA) and cardioembolism (CE) subgroups for prespecified outcomes: 90‐day modified Rankin Scale (mRS) scores of 0–2, 0–3, and 0–4; 90‐day mortality; and symptomatic intracranial hemorrhage (sICH) within 48 h. Estimates were derived as simple effects from models including treatment, etiology, their interaction, age, sex, admission glucose, systolic blood pressure, baseline National Institutes of Health Stroke Scale score, baseline Alberta Stroke Program Early CT Score, intravenous thrombolysis, and occlusion site. Treating center was included as a random intercept for all outcomes except sICH, which was analyzed using Firth penalized logistic regression without center adjustment. *p* values for treatment‐by‐etiology interaction are shown above the brackets. (B) Adjusted ORs with 95% CIs for LAA versus CE for the primary outcome of 90‐day mRS 0–3 across prespecified subgroups among EVT‐treated patients. Subgroup‐specific models used the EVT‐stratum covariate set, including collateral grade, with the subgroup‐defining variable omitted from adjustment. *p* values for etiology‐by‐subgroup interaction are shown. The reference line denotes an OR of 1. AOR, adjusted odds ratio; ASPECTS, Alberta Stroke Program Early CT Score; CE, cardioembolism; EVT, endovascular thrombectomy; IVT, intravenous thrombolysis; LAA, large‐artery atherosclerosis; mRS, modified Rankin Scale; NIHSS, National Institutes of Health Stroke Scale; sICH, symptomatic intracranial hemorrhage; SMT, standard medical therapy.

Whether the association between EVT and outcome differed by etiology was examined in the overall cohort using models containing treatment, etiology, and their interaction. These analyses used the same modeling framework and pretreatment covariates available in both treatment groups; angiographic collateral grade was not included because it was unavailable in the SMT‐only group. Interaction *p* values were obtained using Wald tests, and etiology‐specific associations of EVT with outcome were derived as simple effects from the fitted models. Results are presented in Figure 2A, Table S4, with additional specifications in Method S4.3. Within the EVT cohort, the etiology–primary outcome association was also examined across predefined subgroups (Figure 2B).

As a complementary exploratory analysis, an etiology‐free pretreatment prognostic score was developed in the EVT cohort using 10‐fold out‐of‐fold cross‐validated regularized logistic regression, with TOAST etiology excluded by design. The score was used to characterize prognostic separation and overlap between LAA and CE rather than as a clinical prediction tool or adjustment variable. Model development and performance are detailed in Method S5 and Table S6, with score distributions shown in Figures S4 and S5.

Within the EVT cohort, block‐based decomposition was used to characterize the change in the LAA–CE association for the primary outcome from the crude to the fully adjusted model. Measured covariate domains comprised demographics, baseline stroke severity, imaging features, metabolic/hemodynamic measures, intravenous thrombolysis, and treating center. Shapley/Lindeman–Merenda–Gold decomposition was used to obtain order‐averaged estimates of each domain's contribution to the crude‐to‐adjusted change in the absolute log‐odds contrast.

**FIGURE 3:**
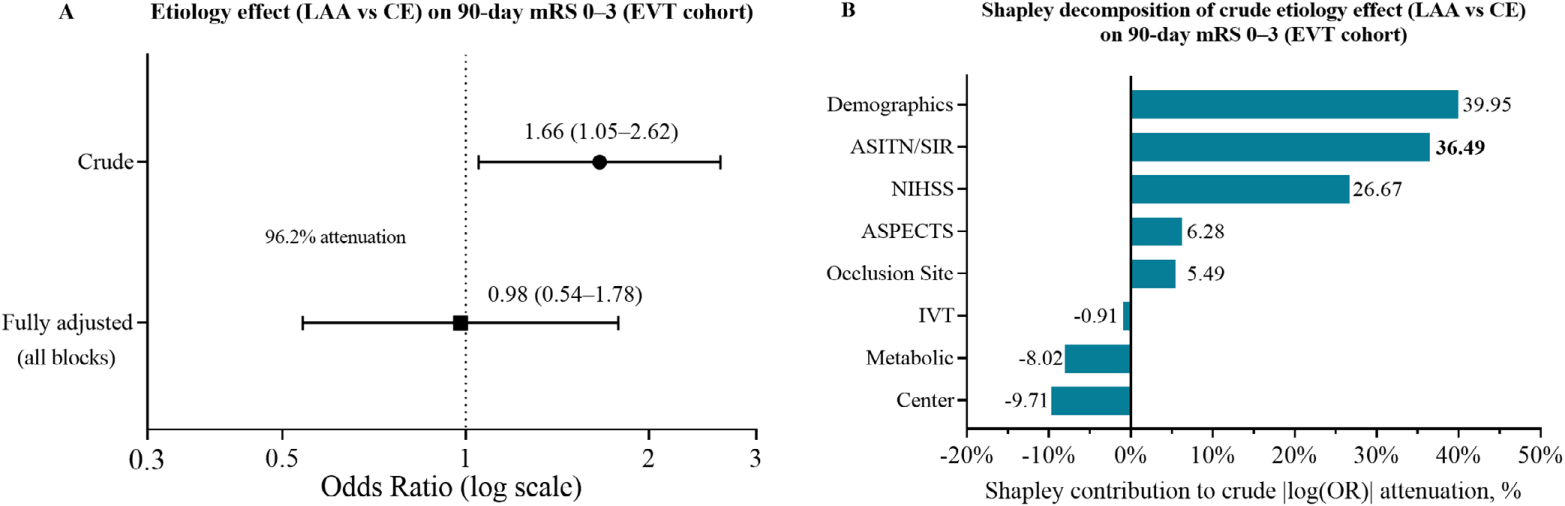
Crude‐to‐adjusted change in the LAA–CE association for 90‐day mRS 0–3 after EVT and domain contributions to that change. (A) Among EVT‐treated patients, LAA was associated with higher crude odds of 90‐day mRS 0–3 than CE (OR, 1.66; 95% CI, 1.05–2.62). After adjustment for the measured covariate domains, the association was close to the null (OR, 0.98; 95% CI, 0.54–1.78). The crude‐to‐adjusted change corresponded to a 96.2% reduction in the absolute log‐odds magnitude of the crude association. (B) Domain contributions to the crude‐to‐adjusted change shown in Panel A were quantified using Shapley/Lindeman–Merenda–Gold decomposition. Signed contributions are expressed as percentage‐point contributions to the overall attenuation. Negative contributions indicate rebound or suppressor‐like behavior that may reflect correlations among domains and odds‐ratio noncollapsibility, rather than protective effects. ASPECTS, Alberta Stroke Program Early CT Score; ASITN/SIR, American Society of Interventional and Therapeutic Neuroradiology/Society of Interventional Radiology collateral grade; CE, cardioembolism; EVT, endovascular thrombectomy; IVT, intravenous thrombolysis; LAA, large‐artery atherosclerosis; NIHSS, National Institutes of Health Stroke Scale; OR, odds ratio.

Fixed‐effects logistic regression with treating center represented by indicator variables was used for the decomposition module. This parameterization supported the sequential, Shapley, and domain‐omission analyses and differed from the random‐intercept structure used in the primary outcome models. Accordingly, the fully adjusted estimate from the decomposition model was interpreted within this analytic framework rather than as a numerical replication of the primary adjusted estimate. The decomposition characterized the statistical contribution of measured covariate domains to the observed crude‐to‐adjusted attenuation and was not intended to estimate causal mediation. Primary decomposition findings are presented in Figure 3, Table 3; sequential, Shapley, domain‐omission, and imaging‐component analyses are detailed in Method S6, Tables S7‐0–S7‐5, Figures S6–S9.

**TABLE 3:** Domain contributions to the crude‐to‐adjusted change in the LAA–CE association with 90‐day mRS 0–3 among EVT‐treated patients.

Covariate domain	Signed Shapley contribution to the overall reduction, percentage points^a^	OR for LAA vs. CE from the domain‐omitted model	Leave‐one‐domain‐out change in absolute log(OR)^b^
Imaging features (ASPECTS, ASITN/SIR collateral grade, and occlusion site)	50.4	1.321	0.259
Demographics (age and sex)	37.0	1.235	0.192
Stroke severity (baseline NIHSS)	26.0	1.150	0.120
Metabolic/BP (glucose and SBP)	−6.9	0.883	0.106
Intravenous thrombolysis	−0.6	0.974	0.007
Treating center (fixed‐effect indicators)	−9.6	0.946	0.036

*Note:* Within the decomposition model, the crude OR for LAA versus CE was 1.659 and the fully adjusted OR was 0.981, corresponding to a 96.2% reduction in the absolute log‐odds magnitude of the crude association.

Abbreviations: ASITN/SIR, American Society of Interventional and Therapeutic Neuroradiology/Society of Interventional Radiology; ASPECTS, Alberta Stroke Program Early CT Score; CE, cardioembolism; EVT, endovascular thrombectomy; LAA, large‐artery atherosclerosis; mRS, modified Rankin Scale; NIHSS, National Institutes of Health Stroke Scale; OR, odds ratio; SBP, systolic blood pressure.

^a^ Shapley/Lindeman–Merenda–Gold contributions were estimated across 2000 randomly sampled domain‐entry orders and are expressed as signed percentage‐point contributions to the overall 96.2% reduction. Positive values indicate movement of the LAA–CE association toward the null, whereas negative values indicate movement away from the null and may arise from correlations among domains or odds‐ratio noncollapsibility. Signed contributions sum to the overall reduction, with minor differences due to rounding.

^b^ Domain‐omitted models were obtained by refitting the fully adjusted decomposition model after excluding the specified domain. The leave‐one‐domain‐out change in absolute log(OR) is calculated relative to the fully adjusted model. Likelihood‐ratio statistics, degrees of freedom, and *p* values are reported in Table S7‐3.

Robustness was further assessed using multiple imputation, alternative mRS dichotomization thresholds, proportional‐odds diagnostics, cutpoint‐specific mixed‐effects logistic models, and marginal standardized estimates on the risk‐difference and risk‐ratio scales. These analyses are detailed in Methods S3, S4.4, and S6, Tables S3, S5, and S7‐6, Figures S1–S3.

## 3 Results

### 3.1 Study Cohort and Admission Phenotype

Among 750 screened patients, 655 were eligible after excluding 5 without 90‐day follow‐up and 90 with etiologies other than LAA or CE (undetermined etiology, *n* = 65; other determined etiologies, *n* = 25) (Figure 1). The eligible cohort included 269 patients with LAA and 386 with CE; 423 received EVT plus SMT and 232 received SMT alone. After excluding 24 patients with missing prespecified covariates, the complete‐case cohort comprised 631 patients (EVT, *n* = 404; SMT alone, *n* = 227; LAA, *n* = 262; CE, *n* = 369).

Admission profiles differed between LAA and CE in both treatment strata (Table 1). In the EVT cohort, patients with LAA were younger, more often male, had lower baseline NIHSS scores, more favorable collateral status, and a substantially lower prevalence of atrial fibrillation than those with CE. Differences in age, stroke severity, ASPECTS, and occlusion pattern were also present in the SMT‐only cohort. EVT‐treated and SMT‐only patients additionally differed in selected admission characteristics, including ASPECTS, systolic blood pressure, and occlusion‐site distribution. Procedural and angiographic characteristics among EVT‐treated patients are reported in Table S1.

### 3.2 Etiology and Outcomes Within Treatment Strata

Among EVT‐treated patients, LAA was associated with higher crude odds of 90‐day mRS 0–3 than CE (OR, 1.59; 95% CI, 1.05–2.42). After adjustment for measured pretreatment characteristics, the estimate moved close to the null (aOR, 0.92; 95% CI, 0.53–1.59) (Table 2). The mortality estimate changed similarly, from an OR of 0.65 (95% CI, 0.43–0.99) before adjustment to an aOR of 0.99 (95% CI, 0.58–1.71).

A similar pattern was observed in the SMT‐only stratum. The LAA–CE association for mRS 0–3 changed from an OR of 2.00 (95% CI, 1.00–3.97) to an aOR of 1.12 (95% CI, 0.47–2.65). The corresponding mortality estimates were 0.42 (95% CI, 0.25–0.71) and 0.54 (95% CI, 0.28–1.02).

### 3.3 Admission Phenotype and the LAA–CE Contrast

In the EVT decomposition model, the LAA–CE association for mRS 0–3 changed from a crude OR of 1.66 to a fully adjusted OR of 0.98. This corresponded to a 96.2% reduction in the absolute log‐odds magnitude of the crude association (Figure 3, Table 3).

The largest positive signed contributions to this change were from imaging features (50.4 percentage points), demographics (37.0 percentage points), and baseline stroke severity (26.0 percentage points), partly offset by negative contributions from other domains. Within the imaging domain, collateral status contributed most (36.5 percentage points), whereas ASPECTS (6.3 percentage points) and occlusion site (5.5 percentage points) made smaller contributions. Full decomposition results are provided in Tables S7‐0–S7‐5.

### 3.4 EVT–Outcome Associations Across Etiologic Subtypes

In the complete‐case cohort, 90‐day mRS 0–3 was achieved by 152 of 404 EVT‐treated patients (37.6%) and 44 of 227 patients receiving SMT alone (19.4%). Mortality occurred in 168 of 404 EVT‐treated patients (41.6%) and 107 of 227 SMT‐only patients (47.1%).

The adjusted EVT–outcome association was similar in magnitude across etiologies. Compared with SMT alone, EVT was associated with higher adjusted odds of mRS 0–3 in both LAA (aOR, 2.94; 95% CI, 1.51–5.73) and CE (aOR, 3.04; 95% CI, 1.46–6.36), with no evidence of treatment‐by‐etiology interaction (P for interaction = 0.944) (Figure 2A; Table S4). Analyses of ordinal mRS shift, alternative functional thresholds, mortality, and sICH likewise showed no consistent heterogeneity by etiology. Within the EVT cohort, adjusted LAA–CE associations for the primary outcome were broadly consistent across prespecified subgroups (Figure 2B).

### 3.5 Secondary and Complementary Analyses

At the more stringent secondary mRS 0–2 threshold, the adjusted LAA–CE estimate favored CE among EVT‐treated patients (aOR, 0.46; 95% CI, 0.25–0.84), whereas the corresponding estimate in the SMT‐only cohort was imprecise (aOR, 0.74; 95% CI, 0.23–2.37). Adjusted analyses of mRS 0–4 and ordinal mRS shift showed no clear LAA–CE differences in either treatment stratum.

Multiple‐imputation analyses yielded estimates similar in direction and magnitude to the complete‐case findings. Proportional‐odds diagnostics indicated nonproportionality globally and for the treatment term, but not for the treatment‐by‐etiology interaction. Cutpoint‐specific models showed no consistent interaction across mRS thresholds (Tables S3 and S5; Figure S3). On marginal risk scales, the standardized probabilities of 90‐day mRS 0–3 were 37.5% for CE and 37.2% for LAA (risk difference, −0.3 percentage points; 95% CI, −9.7 to 9.1; risk ratio, 0.99; 95% CI, 0.76–1.25), consistent with the near‐null adjusted association (Table S7‐6).

The etiology‐free pretreatment prognostic score showed good discrimination for 90‐day mRS 0–3 (out‐of‐fold AUC, 0.794; 95% CI, 0.749–0.833). Predicted probabilities of 90‐day mRS 0–3 were higher on average in LAA than in CE (SMD, 0.453), but the two distributions overlapped substantially (80.8% overlap) (Table S6; Figures S4 and S5).

## 4 Discussion

This study reframes the apparent prognostic contrast between LAA and CE in large‐core anterior‐circulation LVO. The crude functional‐outcome advantage associated with LAA was markedly attenuated after adjustment for admission clinical‐imaging phenotype, yielding a near‐null estimate. In the decomposition framework, this corresponded to a 96.2% reduction in the absolute log‐odds magnitude of the crude association. EVT–outcome associations were similar in magnitude across etiologic subtypes, with no evidence of treatment‐by‐etiology interaction. Taken together, these findings clarify the hyperacute prognostic role of TOAST etiology in large‐core stroke: much of the apparent prognostic signal associated with etiologic subtype is captured by measurable admission clinical‐imaging phenotype, which is more directly relevant to prognostic assessment and EVT selection.

Prior EVT cohorts have reported inconsistent associations between TOAST etiology and outcome. The SITS registry found worse adjusted outcomes in LAA than in CE [12], whereas other multicenter studies reported broadly similar 90‐day outcomes across etiologic subtypes [16]. A plausible explanation is that etiologic subtype is closely coupled to admission phenotype. LAA and CE often differ in demographic profile, collateral recruitment, baseline neurologic severity, and occlusion characteristics, all of which are established prognostic factors in acute LVO [12, 17, 18, 25, 26, 27]. Differences in how these features are captured and modeled can therefore materially alter the apparent LAA–CE association across cohorts [13, 28]. Because patients with large‐core infarction have substantial established tissue injury and limited residual tissue reserve, findings from broader or non‐large‐core EVT cohorts cannot be assumed to apply directly to this clinically distinct population. This issue is particularly consequential in large‐core LVO, where prognosis and hyperacute EVT selection are strongly anchored to baseline clinical and imaging severity. The present study advances prior work by quantifying not only whether the crude etiologic contrast persisted after adjustment but also how much it changed and which domains contributed most. Imaging features, particularly collateral status, accounted for the largest share of the crude‐to‐adjusted attenuation, followed by demographics and baseline NIHSS. These findings support the interpretation that the apparent prognostic signal associated with TOAST subtype in large‐core stroke is largely embedded within the admission phenotype rather than carried by the etiologic label alone. The decomposition characterizes model‐based statistical attenuation and does not estimate causal mediation [28, 29].

The adjusted associations between EVT and outcomes were similar in LAA and CE, with no evidence of treatment‐by‐etiology interaction. At the more stringent mRS 0–2 threshold, however, CE was associated with higher adjusted odds of functional independence among EVT‐treated patients. Functional independence was relatively uncommon in this large‐core cohort, and the number of patients achieving this outcome—particularly in the LAA group—was modest. Because this was a secondary, threshold‐specific signal that was not reproduced across the ordinal mRS distribution, the broader mRS 0–3 and mRS 0–4 thresholds, or treatment‐by‐etiology interaction analyses, it should be regarded as hypothesis‐generating and warrants confirmation in larger independent cohorts. Overall, these findings support a phenotype‐centered approach to hyperacute EVT selection in large‐core LVO and do not support modifying treatment selection solely on the basis of LAA versus CE etiology. This boundary is temporal, not absolute. After the decision to pursue EVT, suspected intracranial atherosclerotic disease may still inform procedural planning, while definitive etiologic classification remains relevant to subsequent mechanism evaluation and secondary prevention [30, 31].

Several limitations merit consideration. First, because EVT was not randomly assigned, residual confounding and treatment‐selection bias may persist despite adjustment for prespecified clinical and imaging covariates, treating‐center effects, and multiple‐imputation sensitivity analyses; the etiology‐specific EVT estimates therefore represent adjusted associations rather than causal treatment effects. Second, the decomposition quantified model‐based statistical attenuation of the observed LAA–CE association. Prespecified covariate domains, order‐averaged Shapley estimates, and complementary marginal‐risk analyses supported this descriptive interpretation, but the 96.2% reduction should not be interpreted as a causal mediation proportion [28, 32]. The magnitude and allocation of these contributions are conditional on the measured covariates, model specification, and effect scale. Third, although TOAST subtype was independently assessed and disagreements were adjudicated, some etiologic misclassification remains possible [30]. Fourth, the 38‐center Chinese cohort strengthens the relevance of the findings to contemporary practice in China, but external validation is warranted, and the conclusions should not be extended beyond patients with adjudicated LAA or CE. Finally, although etiology‐specific EVT associations were broadly comparable across outcomes and sensitivity analyses, modest heterogeneity, particularly for infrequent outcomes such as sICH, cannot be excluded.

## 5 Conclusion

In large‐core anterior‐circulation LVO, admission clinical‐imaging phenotype captured much of the apparent prognostic signal associated with LAA versus CE, with the adjusted contrast approaching the null. Similar adjusted associations between EVT and outcomes across etiologic subtypes support hyperacute EVT selection based primarily on directly assessed clinical and imaging features rather than on LAA versus CE etiology alone.

## Author Contributions

Rongzong Li: conceptualization, supervision, and writing – review and editing. Wenjie Zi: conceptualization, supervision, funding acquisition, and writing – review and editing. Chengsong Yue: investigation, data curation, formal analysis, and writing – original draft. Xiongxiong Hou and Shihai Yang: investigation and data curation. Jingfu Ma, Xu Xu, Xiaolei Shi, Linyu Li, Jie Yang, Changwei Guo, and Jiaxing Song: investigation.

## Funding

This study was supported by Xinqiao Hospital, Army Medical University, through the Discipline Talent Construction Special Project (Grant No. 2022XKRC003).

## Ethics Statement

The registry protocol was approved by the Ethics Committee of Xinqiao Hospital, Army Medical University (approval No. 2021‐yandi 134‐01) and by the institutional review board of each participating center. The study was conducted in accordance with the Declaration of Helsinki and its subsequent amendments.

## Consent

Written informed consent was obtained from all patients or their legally authorized representatives before enrollment.

## Conflicts of Interest

The authors declare no conflicts of interest.

## Supporting information


**Supplementary Material:** cns71029‐sup‐0001‐supinfo.docx.

## Data Availability

The data that support the findings of this study are available from the corresponding author upon reasonable request. Access to individual‐level participant data is subject to approval by the study investigators and applicable ethical and data‐protection requirements.
