# Supplementary material for: Admission Phenotype Clarifies the Apparent Prognostic Contrast Between TOAST Etiologic Subtypes in Large‐Core Anterior‐Circulation Large‐Vessel Occlusion
Source: CNS Neurosci Ther. 2026 Jul 13;32(7):e71029. doi: 10.1002/cns.71029 (PMC13365358; doi:10.1002/cns.71029)
Supplement: Supplementary file 1 — Supplementary Material: cns71029‐sup‐0001‐supinfo.docx. [file CNS-32-e71029-s001.docx]

**content**

[**Supplementary Methods** 3](#_Toc234161512)

[**Supplementary Method 1. Study Design and Population** 3](#_Toc234161513)

[**Supplementary Method 2. Variables and Definitions** 3](#_Toc234161514)

[**Supplementary Method 3. Missing Data Control** 4](#_Toc234161515)

[**Supplementary Method 4. Statistical Modeling and Adjustment Strategy** 5](#_Toc234161516)

[**Supplementary Method 5. Etiology-Free Pretreatment Prognostic Score (10-Fold Out-of-Fold Evaluation; Complete-Case)** 7](#_Toc234161517)

[**Supplementary Method 6. Block-Wise Decomposition of the LAA–CE Association Among EVT-Treated Patients** 7](#_Toc234161518)

[**Supplementary Table 1. Procedural and workflow characteristics among EVT-treated patients by TOAST etiology (LAA vs CE)** 11](#_Toc234161519)

[**Supplementary Table 2. Associations of Stroke Etiology (LAA vs CE) With Clinical Outcomes in the Complete-Case Cohort, Stratified by Treatment (EVT vs SMT)** 13](#_Toc234161520)

[**Supplementary Table 3. Associations Between Stroke Etiology and Clinical Outcomes Within Treatment Strata: Multiple-Imputation Sensitivity Analysis (m=20)** 16](#_Toc234161521)

[**Supplementary Table 4. Adjusted Associations of Endovascular Thrombectomy With Clinical Outcomes Within Etiologic Subtypes and Etiology-by-Treatment Interactions: Complete-Case and Multiple-Imputation Analyses** 19](#_Toc234161522)

[**Supplementary Table 5. Assessment of the Proportional-Odds Assumption** 21](#_Toc234161523)

[**Supplementary Table 6. Ten-Fold Out-of-Fold Performance of the Etiology-Free Pretreatment Prognostic Score for Favorable 90-Day Functional Outcome (mRS 0–3) in the EVT Cohort** 22](#_Toc234161524)

[**Supplementary Table 7-0. Domain-Level Summary of the Crude-to-Adjusted Change in the LAA–CE Association With 90-Day mRS 0–3 Among EVT-Treated Patients** 23](#_Toc234161525)

[**Supplementary Table 7-1. Sequential Change in the LAA–CE Association With 90-Day mRS 0–3 Among EVT-Treated Patients** 25](#_Toc234161526)

[**Supplementary Table 7-2. Order-Averaged Shapley/LMG Domain Contributions to the Crude-to-Adjusted Change in the LAA–CE Association** 26](#_Toc234161527)

[**Supplementary Table 7-3. Drop-One-Domain Likelihood-Ratio Comparisons and Relative Contributions to Model Fit** 27](#_Toc234161528)

[**Supplementary Table 7-4. Leave-One-Domain-Out Changes in the LAA–CE Association Relative to the Fully Adjusted Model** 28](#_Toc234161529)

[**Supplementary Table 7-5. Order-Averaged Shapley/LMG Contributions After Separating the Imaging Domain Into Collateral Grade, ASPECTS, and Occlusion Site** 29](#_Toc234161530)

[**Supplementary Table 7-6. Marginal Standardized Risks, Risk Difference, and Risk Ratio for 90-Day mRS 0–3 by Etiology** 30](#_Toc234161531)

[**Supplementary Figure legends** 31](#_Toc234161532)

[**Supplementary Figure 1. Missing Data Pattern in Baseline Covariates** 31](#_Toc234161533)

[**Supplementary Figure 2. Diagnostics for Multiple Imputation of Baseline Covariates** 32](#_Toc234161534)

[**Supplementary Figure 3. Adjusted EVT–Outcome Associations Across mRS Thresholds by Stroke Etiology** 34](#_Toc234161535)

[**Supplementary Figure 4. Distribution of Out-of-Fold Predicted Probabilities for Favorable 90-Day Functional Outcome by Etiologic Subtype** 35](#_Toc234161536)

[**Supplementary Figure 5. Empirical Cumulative Distribution of Out-of-Fold Predicted Probabilities for Favorable 90-Day Functional Outcome by Etiologic Subtype** 36](#_Toc234161537)

[**Supplementary Figure 6. Sequential Crude-to-Adjusted Change in the LAA–CE Association Among EVT-Treated Patients** 38](#_Toc234161538)

[**Supplementary Figure 7. Order-Averaged Shapley/LMG Contributions of Covariate Domains to the Crude-to-Adjusted Change in the LAA–CE Association** 39](#_Toc234161539)

[**Supplementary Figure 8. Leave-One-Domain-Out Changes in the LAA–CE Association Relative to the Fully Adjusted Model** 40](#_Toc234161540)

[**Supplementary Figure 9. Order-Averaged Shapley/LMG Contributions After Separating the Imaging Domain** 41](#_Toc234161541)

# **Supplementary Methods**

## **Supplementary Method 1. Study Design and Population**

This study was a secondary analysis of the MAGIC registry, a prospective, multicenter observational cohort that consecutively enrolled patients with acute ischemic stroke due to large-vessel occlusion presenting within 24 hours of symptom onset or last known well^[1]^. For the present analysis, we included adults with anterior-circulation occlusion involving the intracranial internal carotid artery or the M1 or M2 segment of the middle cerebral artery, a large infarct core defined as an ASPECTS ≤5 on baseline noncontrast CT, and stroke etiology classified as large-artery atherosclerosis (LAA) or cardioembolism (CE).

Among 750 screened patients, 5 lacked 90-day follow-up and 90 had etiologies other than LAA or CE (undetermined etiology, n=65; other determined etiologies, n=25), leaving 655 eligible patients. Patients were categorized according to the treatment received: endovascular thrombectomy plus standard medical therapy (EVT, n=423) or standard medical therapy alone (SMT, n=232). The unified complete-case cohort used for adjusted analyses included 631 patients (EVT, n=404; SMT alone, n=227; LAA, n=262; CE, n=369). The study was approved by the institutional review boards of participating centers; consent procedures are reported in the main manuscript. Cohort derivation is shown in Figure 1.

## **Supplementary Method 2.** **Variables and Definitions**

Stroke etiology was classified according to the TOAST framework as described in the main manuscript. The present analysis was restricted to patients classified as large-artery atherosclerosis (LAA) or cardioembolism (CE).^[2]^

Pretreatment clinical variables included age, sex, admission National Institutes of Health Stroke Scale (NIHSS) score,^[3]^ admission glucose, systolic blood pressure (SBP), and intravenous thrombolysis (IVT) status. Imaging variables included baseline Alberta Stroke Program Early CT Score (ASPECTS; range, 0–10)^[4]^ and occlusion site, categorized as intracranial internal carotid artery, M1 segment, or M2 segment.

Among EVT-treated patients, collateral status was assessed on pre-reperfusion angiography using the American Society of Interventional and Therapeutic Neuroradiology/Society of Interventional Radiology (ASITN/SIR) collateral grading scale^[5]^ and categorized as 0–1, 2, or 3–4. Angiographic collateral grade was unavailable in the SMT-only cohort and was therefore included only in EVT-stratum models.

EVT workflow and procedural variables, including onset-to-puncture time, puncture-to-final angiographic assessment time, first-line thrombectomy technique, number of device passes, and final reperfusion grade, were summarized descriptively in Supplementary Table 1 and were not included as adjustment covariates. Successful reperfusion was defined as a modified Thrombolysis in Cerebral Infarction (mTICI) grade of 2b–3.^[6]^

The primary outcome was a favorable functional outcome, defined as a 90-day modified Rankin Scale (mRS) score of 0–3.^[7]^ Secondary efficacy outcomes were 90-day mRS 0–2, mRS 0–4, and ordinal shift across the full 90-day mRS distribution (0–6). Safety outcomes were 90-day all-cause mortality and symptomatic intracranial hemorrhage (sICH) within 48 hours, defined according to the Heidelberg Bleeding Classification.^[8]^

## **Supplementary Method 3. Missing Data Control**

Overall missingness in baseline covariates was low. Primary analyses therefore used a complete-case approach. A unified complete-case cohort was defined by requiring nonmissing data on treatment, etiology, treating center, all selected pretreatment covariates, and all analyzed outcomes, allowing the same participants to be used across adjusted outcome analyses (n=631; EVT, n=404; SMT alone, n=227; LAA, n=262; CE, n=369). Patterns and proportions of missing baseline covariates are shown in Supplementary Figure 1.

As a sensitivity analysis, missing baseline covariates were handled using multiple imputation by chained equations (MICE; mice package in R).^[9, 10]^ The imputation model included treatment, etiology, treating center, all selected pretreatment covariates, and the analyzed outcomes. Treatment, etiology, and outcomes were not imputed. Twenty imputed datasets were generated with 10 iterations per dataset using a fixed random seed (12345), and model estimates were combined using Rubin’s rules.^[9, 11]^ Imputation diagnostics are shown in Supplementary Figure 2.

**Supplementary Method 4. Statistical Modeling and Adjustment Strategy**

**4.1 Outcomes and Effect Measures**

Binary outcomes—90-day mRS 0–2, mRS 0–3, mRS 0–4, 90-day all-cause mortality, and symptomatic intracranial hemorrhage (sICH) within 48 hours—were analyzed using logistic regression and are reported as odds ratios (ORs) with 95% confidence intervals (CIs). The ordinal 90-day mRS distribution (0–6) was analyzed using proportional-odds regression and is reported as a common OR (cOR) with 95% CIs.

Binary functional outcomes were coded as 1 for a favorable outcome; therefore, an OR greater than 1 indicates higher odds of a favorable outcome for the modeled comparison. Mortality and sICH were coded as 1 for the occurrence of the event; therefore, an OR less than 1 indicates lower odds of the adverse outcome. The ordinal mRS scale was parameterized such that a cOR greater than 1 indicates a shift toward lower, more favorable mRS scores.

**4.2 Etiology–Outcome Associations Within Treatment Strata**

Associations between stroke etiology and outcomes were evaluated separately among EVT-treated patients and patients receiving SMT alone, with CE as the reference category.

In the EVT-treated stratum, adjusted models included age, sex, admission glucose, systolic blood pressure, baseline NIHSS score, baseline ASPECTS, intravenous thrombolysis status, occlusion site, and angiographic collateral grade categorized as ASITN/SIR 0–1, 2, or 3–4. Treating center was modeled as a random intercept.

In the SMT-only stratum, adjusted models included age, sex, admission glucose, systolic blood pressure, baseline NIHSS score, baseline ASPECTS, intravenous thrombolysis status, and occlusion site. Angiographic collateral grade was not included because it was unavailable in patients receiving SMT alone. Treating center was modeled as a random intercept.

Because sICH events were sparse, crude and adjusted associations with sICH were estimated using Firth penalized logistic regression without a center random effect.^[12]^ Selected complete-case results are presented in Table 2, with complete outcome estimates in Supplementary Table 2. Multiple-imputation sensitivity analyses are presented in Supplementary Table 3.

**4.3 EVT–Outcome Association, Etiology-by-Treatment Interaction, and Etiology-Specific Estimates**

Whether the association between EVT and outcome differed by etiology was evaluated in the overall cohort using models containing treatment (EVT vs SMT alone; reference, SMT alone), etiology (LAA vs CE; reference, CE), and a treatment-by-etiology interaction term. Interaction P values were obtained using Wald tests.

Models adjusted for age, sex, admission glucose, systolic blood pressure, baseline NIHSS score, baseline ASPECTS, intravenous thrombolysis status, and occlusion site. Angiographic collateral grade was not included because it was unavailable in the SMT-only group.

Binary outcomes were analyzed using mixed-effects logistic regression, and ordinal mRS shift was analyzed using mixed-effects proportional-odds regression, with treating center modeled as a random intercept. Because sICH events were sparse, Firth penalized logistic regression without center adjustment was used for this outcome.

Etiology-specific associations of EVT with outcome were derived as simple effects from the fitted interaction models. Complete-case and multiple-imputation estimates for the etiology-specific EVT–outcome associations and treatment-by-etiology interactions are presented in Supplementary Table 4.

**4.4 Assessment of the Proportional-Odds Assumption and Cutpoint-Specific Sensitivity Analyses**

For ordinal mRS analyses, the proportional-odds assumption was evaluated using a Brant-type diagnostic based on a standard proportional-odds model without a center term,^[13]^ used for diagnostic purposes only (Supplementary Table 5). As a sensitivity analysis, cutpoint-specific associations were estimated using a sequence of binary mixed-effects logistic regression models at each mRS threshold (mRS ≤k vs >k), with treating center modeled as a random intercept when model convergence was achieved (Supplementary Figure 3).

**Supplementary Method 5. Etiology-Free Pretreatment Prognostic Score (10-Fold Out-of-Fold Evaluation; Complete-Case)**

**Objective.** To contextualize the etiologic comparisons, we constructed an etiology-free prognostic score using selected pretreatment clinical and imaging characteristics, with TOAST etiology excluded by design. This complementary analysis characterized prognostic separation and overlap between LAA and CE.

**Endpoint.** The endpoint was favorable 90-day functional outcome, defined as an mRS score of 0–3.

**Sample.** The analysis was performed in the EVT cohort and included 423 patients with available outcome data and complete information for the predictors listed below.

**Predictors.** Predictors included age, sex, admission glucose, baseline NIHSS score, baseline ASPECTS, occlusion site (intracranial internal carotid artery, M1, or M2), intravenous thrombolysis status, angiographic collateral grade (ASITN/SIR 0–1, 2, or 3–4), and treating center. TOAST etiology was not included. Because the regularized regression model did not accommodate random effects, treating center was represented using indicator variables.

**Modeling and evaluation.** Penalized logistic regression was fitted using the glmnet package with 10-fold cross-validation.^[14]^ Out-of-fold (OOF) predicted probabilities were obtained for each participant and used as the etiology-free prognostic score. The OOF area under the receiver operating characteristic curve was 0.794 (95% CI, 0.749–0.833; Supplementary Table 6). Predicted probabilities were higher on average in LAA than in CE (SMD, 0.453), while the two distributions showed substantial overlap, quantified using a distribution-free overlapping index (80.8%; Kolmogorov–Smirnov P=1.07×10⁻⁴; Supplementary Figures 4 and 5).^[15]^

**Supplementary Method 6. Block-Wise Decomposition of the LAA–CE Association Among EVT-Treated Patients**

**6.1 Objective and Analytic Framework**

Within the EVT cohort, we examined how the observed association between stroke etiology and favorable 90-day functional outcome changed after accounting for measured covariate domains. The outcome was a 90-day modified Rankin Scale (mRS) score of 0–3, and etiology was modeled as large-artery atherosclerosis (LAA) versus cardioembolism (CE), with CE as the reference category.

Decomposition analyses used fixed-effects logistic regression, with treating center represented by indicator variables. Covariate domains were defined a priori for the present analysis and were applied consistently across the sequential, Shapley, and domain-omission analyses.

**6.2 Covariate Domains**

The covariate domains were:

- **Demographics:** age and sex
- **Stroke severity:** baseline NIHSS score
- **Imaging features:** baseline ASPECTS, angiographic ASITN/SIR collateral grade, and occlusion site
- **Metabolic/hemodynamic measures:** admission glucose and systolic blood pressure
- **Intravenous thrombolysis:** IVT status
- **Treating center:** center indicator variables

For the sequential analysis, domains were entered in the following predefined order: demographics, stroke severity, imaging features, metabolic/hemodynamic measures, intravenous thrombolysis, and treating center.

**6.3 Sequential and Shapley Decomposition Analyses**

Sequential models were fitted beginning with the crude model containing etiology alone, followed by addition of the covariate domains in the predefined order. At each step, we recorded the OR for LAA versus CE with its 95% CI and P value, together with the cumulative reduction in the absolute log-odds magnitude of the crude association and the incremental change from the preceding model. Positive incremental values indicate movement of the LAA–CE association toward the null, whereas negative values indicate movement away from the null. Sequential results are presented in Supplementary Table 7-1 and Supplementary Figure 6.

Because sequential results depend on the order in which domains enter the model, Shapley/Lindeman–Merenda–Gold decomposition was used to estimate domain contributions averaged across 2,000 randomly sampled domain-entry orders using a fixed random seed.^[16, 17]^ For each ordering, the marginal change in the absolute log-odds magnitude of the LAA–CE association was recorded when a domain entered the model. These marginal changes were averaged across sampled orderings.

Shapley results are reported as signed percentage-point contributions to the overall crude-to-adjusted attenuation. Positive contributions indicate that a domain moved the LAA–CE association toward the null on average, whereas negative contributions indicate movement away from the null. Domain-level contributions are reported in Table 3 and Supplementary Table 7-2 and displayed in Supplementary Figure 7.

**6.4 Complementary Domain Analyses**

To assess the contribution of each domain to overall model fit, the full model was compared with models omitting one domain at a time using likelihood-ratio tests. The relative loss in model fit after omission of each domain was summarized in Supplementary Table 7-3. This analysis evaluates the outcome information carried by each domain and is distinct from its contribution to attenuation of the LAA–CE association.

We also re-estimated the LAA–CE association after removing each domain individually from the fully adjusted model. For each domain-omitted model, we report the OR for LAA versus CE and the change in absolute log-odds magnitude relative to the full model. A larger positive change indicates that omission of the domain moved the association farther from the null. Results are reported in Supplementary Table 7-4 and displayed in Supplementary Figure 8.

To further characterize the imaging contribution, the combined imaging domain was separated into angiographic ASITN/SIR collateral grade, baseline ASPECTS, and occlusion site. The Shapley decomposition was then repeated with these imaging components entered as separate domains. Results are reported in Supplementary Table 7-5 and Supplementary Figure 9.

**6.5 Marginal Standardization Sensitivity Analysis**

As a sensitivity analysis on marginal risk scales, standardized risks for the primary outcome were estimated from the fully adjusted fixed-effects logistic regression model used in the decomposition analysis, including treating-center indicator variables. For each participant, predicted probabilities of 90-day mRS 0–3 were generated twice by setting etiology to LAA and CE, respectively, while retaining all measured covariates at their observed values. The predicted probabilities were then averaged across participants to obtain the marginal standardized risk under each etiologic category.^[18]^

The risk difference was calculated as the standardized risk for LAA minus that for CE, and the risk ratio was calculated as the standardized risk for LAA divided by that for CE. Confidence intervals for the standardized risks, risk difference, and risk ratio were estimated using nonparametric bootstrap resampling with 1,000 replicates.^[19]^ Results are reported in Supplementary Table 7-6.

**6.6 Statistical Reporting**

ORs, 95% CIs, and P values were Wald-based unless otherwise specified. Likelihood-ratio tests were used for comparisons between nested models. Treating center was represented by fixed indicator variables in the decomposition analyses to support domain-level decomposition and nested model comparisons; the primary outcome models instead used a random intercept for treating center.

The decomposition characterizes how measured covariate domains contributed to the observed crude-to-adjusted change in the LAA–CE association. It was not used to estimate causal mediation.

Abbreviations

IVT, intravenous thrombolysis; ASITN/SIR, angiographic collateral grade; LMG, Lindeman–Merenda–Gold.

**Supplementary Tables**

## **Supplementary Table 1. Procedural and workflow characteristics among EVT-treated patients by TOAST etiology (LAA vs CE)**

|  |  | | LAA stratum | CE stratum |  |
| --- | --- | --- | --- | --- | --- |
|  |  | | EVT group (n=143) | EVT group (n=261) | *SMD* |
| **Procedural characteristics (EVT only)** | | |  |  |  |
| First-line thrombectomy approach | | |  |  | 0.589 |
|  | stent thrombectomy | | 63 (44.1) | 82 (31.4) |  |
|  | aspiration | | 56 (39.2) | 170 (65.1) |  |
|  | others | | 24 (16.8) | 9 (3.4) |  |
| OTP, min, median (IQR) | | | 405.0 [259.5, 602.5] | 340.0 [240.0, 510.0] | 0.214 |
| PT, min, median (IQR) | | | 513.0 [362.0, 742.5] | 427.0 [316.0, 598.0] | 0.344 |
| Anesthesia, general, n (%) | | | 28 (19.6) | 42 (16.1) | 0.091 |
| mTICI | | |  |  | 0.126 |
|  | | 0-2a | 22 (15.1) | 32 (11.6) |  |
|  | | 2b-3 | 121 (84.6) | 232 (88.9) |  |
| Continuous variables are presented as median [IQR], and categorical variables as n (%).  This table is restricted to EVT-treated patients and summarizes procedural and workflow characteristics by TOAST etiology (LAA vs CE) in the complete-case cohort.  Standardized mean differences (SMDs) quantify between-etiology imbalance (absolute SMD values of ~0.1, ~0.2, and ~0.5 are often interpreted as small, moderate, and large differences, respectively).  Successful reperfusion was defined as mTICI ≥2b.  PT was defined as puncture-to-final angiographic assessment time (mTICI ≥2b time for successful reperfusion; otherwise the final angiogram/procedure termination time).  The first-line thrombectomy approach was categorized as stent retriever, aspiration, or others (including combined techniques).  Abbreviations: LAA, large-artery atherosclerosis; CE, cardioembolism; OTP, onset-to-puncture time; PT, puncture-to-final angiographic assessment time; mTICI, modified Thrombolysis in Cerebral Infarction. | | | | | |

## **Supplementary Table 2. Associations of Stroke Etiology (LAA vs CE) With Clinical Outcomes in the Complete-Case Cohort, Stratified by Treatment (EVT vs SMT)**

| Patients treated with EVT | | | | | | | | | |
| --- | --- | --- | --- | --- | --- | --- | --- | --- | --- |
|  | | CE population  (n=261) | LAA population (n=143) | Crude OR  95 % CI | p-value | Model 1 | | Model 2 | |
|  |  |  |  |  |  | Adjusted OR  95 % CI | p-value | Adjusted OR  95 % CI | p-value |
| Primary outcome | |  |  |  |  |  |  |  |  |
|  | mRS 0-3 | 88 (33.7) | 64 (44.8) | 1.59 [1.05, 2.42] | 0.029 | 0.90 [0.53, 1.54] | 0.706 | 0.92 [0.53, 1.59] | 0.763 |
| Secondary outcome | |  |  |  |  |  |  |  |  |
|  | mRS 0-2 | 62 (23.8) | 28 (19.6) | 0.78 [0.47, 1.29] | 0.336 | 0.47 [0.26, 0.85] | 0.012 | 0.46 [0.25, 0.84] | 0.012 |
|  | mRS 0-4 | 125 (47.9) | 79 (55.2) | 1.34 [0.89, 2.02] | 0.158 | 0.73 [0.43, 1.24] | 0.245 | 0.73 [0.43, 1.24] | 0.245 |
|  | Ordinal mRS shift (0–6) | 5 [3, 6] | 4 [3, 6] | 1.30 [0.90, 1.85] | 0.166 | 0.81 [0.53, 1.22] | 0.31 | 0.82 [0.54, 1.25] | 0.354 |
| Safety outcome | |  |  |  |  |  |  |  |  |
|  | Mortality | 118 (45.2) | 50 (35.0) | 0.65 [0.43, 0.99] | 0.046 | 1.03 [0.61, 1.74] | 0.91 | 0.99 [0.58, 1.71] | 0.98 |
|  | sICH | 35 (13.4) | 15 (10.5) | 0.77 [0.40, 1.43] | 0.413 | 0.99 [0.48, 1.96] | 0.971 | NA | NA |
| Patients treated with SMT only | | | | | | | | | |
|  | | CE population  (n=108) | LAA population (n=119) | Crude OR  95 % CI | p-value | Model 3 | | Model 4 | |
|  |  |  |  |  |  | Adjusted OR  95 % CI | p-value | Adjusted OR  95 % CI | p-value |
| Primary outcome | |  |  |  |  |  |  |  |  |
|  | mRS 0-3 | 15 (13.9) | 29 (24.4) | 2.00 [1.00, 3.97] | 0.048 | 1.07 [0.47, 2.42] | 0.873 | 1.12 [0.47, 2.65] | 0.795 |
| Secondary outcome | |  |  |  |  |  |  |  |  |
|  | mRS 0-2 | 7 (6.5) | 12 (10.1) | 1.62 [0.61, 4.27] | 0.331 | 0.79 [0.26, 2.42] | 0.676 | 0.74 [0.23, 2.37] | 0.614 |
|  | mRS 0-4 | 30 (27.8) | 59 (49.6) | 2.56 [1.47, 4.45] | <0.001 | 1.55 [0.80, 2.99] | 0.19 | 1.58 [0.81, 3.08] | 0.183 |
|  | Ordinal mRS shift (0–6) | 6 [4, 6] | 5 [4, 6] | 2.33 [1.41, 3.85] | <0.001 | 1.33 [0.77, 2.33] | 0.304 | 1.35 [0.77, 2.38] | 0.296 |
| Safety outcome | |  |  |  |  |  |  |  |  |
|  | Mortality | 63 (58.3) | 44 (37.0) | 0.42 [0.25, 0.71] | 0.001 | 0.56 [0.30, 1.04] | 0.067 | 0.54 [0.28, 1.02] | 0.059 |
|  | sICH | 3 (2.8) | 3 (2.5) | 0.91 [0.19, 4.36] | 0.897 | 1.09 [0.16, 7.69] | 0.931 | NA | NA |
| Data are presented as number (percentage) for binary outcomes and median (interquartile range) for the ordinal modified Rankin Scale distribution. Odds ratios (ORs) and 95% confidence intervals (CIs) compare LAA with CE, with CE as the reference category, within each treatment stratum. For favorable functional outcomes (90-day mRS 0–2, mRS 0–3, and mRS 0–4), an OR greater than 1 indicates higher odds of a favorable outcome with LAA. For mortality and sICH, which were coded as event=1, an OR less than 1 indicates lower odds of the adverse event with LAA. For the ordinal mRS analysis, the common OR (cOR) was parameterized such that a value greater than 1 indicates a shift toward lower, more favorable mRS scores with LAA.  Analyses were conducted separately among EVT-treated patients and patients receiving SMT alone. All adjusted models included pretreatment covariates only; procedural and post-treatment variables were not included.  In the EVT-treated stratum (n=404; CE, n=261; LAA, n=143), Model 1 adjusted for age, sex, admission glucose, systolic blood pressure, baseline NIHSS score, baseline ASPECTS, intravenous thrombolysis status, occlusion site, and angiographic ASITN/SIR collateral grade. Model 2 included the same covariates and additionally modeled treating center as a random intercept using mixed-effects logistic regression for binary outcomes and mixed-effects proportional-odds regression for ordinal mRS shift.  In the SMT-only stratum (n=227; CE, n=108; LAA, n=119), Model 3 adjusted for age, sex, admission glucose, systolic blood pressure, baseline NIHSS score, baseline ASPECTS, intravenous thrombolysis status, and occlusion site. Model 4 included the same covariates and additionally modeled treating center as a random intercept using mixed-effects logistic regression for binary outcomes and mixed-effects proportional-odds regression for ordinal mRS shift. Angiographic collateral grade was not included because it was unavailable in the SMT-only group.  Because sICH events were sparse, crude and covariate-adjusted estimates for sICH were obtained using Firth penalized logistic regression without a center random effect. sICH was defined according to the Heidelberg Bleeding Classification.  Abbreviations: ASITN/SIR, American Society of Interventional and Therapeutic Neuroradiology/Society of Interventional Radiology collateral grade; ASPECTS, Alberta Stroke Program Early CT Score; CE, cardioembolism; CI, confidence interval; cOR, common odds ratio; EVT, endovascular thrombectomy; IQR, interquartile range; IVT, intravenous thrombolysis; LAA, large-artery atherosclerosis; mRS, modified Rankin Scale; NIHSS, National Institutes of Health Stroke Scale; OR, odds ratio; sICH, symptomatic intracranial hemorrhage; SMT, standard medical therapy. | | | | | | | | | |

## **Supplementary Table 3. Associations Between Stroke Etiology and Clinical Outcomes Within Treatment Strata: Multiple-Imputation Sensitivity Analysis (m=20)**

| Patients treated with EVT | | | | | | | | | |
| --- | --- | --- | --- | --- | --- | --- | --- | --- | --- |
|  | | CE population  (n=277) | LAA population (n=146) | Crude OR  95 % CI | p-value | Model 1 | | Model 2 | |
|  |  |  |  |  |  | Adjusted OR  95 % CI | p-value | Adjusted OR  95 % CI | p-value |
| Primary outcome | |  |  |  |  |  |  |  |  |
|  | mRS 0-3 | 92 (33.2) | 66 (45.2) | 1.66 [1.10, 2.50] | 0.016 | 0.95 [0.56, 1.60] | 0.836 | 0.96 [0.56, 1.65] | 0.881 |
| Secondary outcome | |  |  |  |  |  |  |  |  |
|  | mRS 0-2 | 64 (23.1) | 30 (20.5) | 0.86 [0.53, 1.40] | 0.548 | 0.48 [0.27, 0.87] | 0.016 | 0.47 [0.26, 0.87] | 0.015 |
|  | mRS 0-4 | 129 (46.6) | 82 (56.2) | 1.47 [0.98, 2.20] | 0.061 | 0.82 [0.49, 1.39] | 0.464 | 0.82 [0.49, 1.39] | 0.464 |
|  | Ordinal mRS shift (0–6) | 5 [3, 6] | 4 [3, 6] | 1.39 [0.96, 1.96] | 0.077 | 0.81 [0.54, 1.23] | 0.326 | 0.82 [0.54, 1.25] | 0.356 |
| Safety outcome | |  |  |  |  |  |  |  |  |
|  | Mortality | 128 (46.2) | 50 (34.2) | 0.61 [0.40, 0.92] | 0.018 | 0.96 [0.57, 1.61] | 0.867 | 0.92 [0.53, 1.59] | 0.769 |
|  | sICH | 38 (13.7) | 15 (10.3) | 0.73 [0.39, 1.37] | 0.332 | 0.95 [0.49, 1.87] | 0.885 | NA | NA |
| Patients treated with SMT only | | | | | | | | | |
|  | | CE population  (n=109) | LAA population (n=123) | Crude OR  95 % CI | p-value | Model 3 | | Model 4 | |
|  |  |  |  |  |  | Adjusted OR  95 % CI | p-value | Adjusted OR  95 % CI | p-value |
| Primary outcome | |  |  |  |  |  |  |  |  |
|  | mRS 0-3 | 15 (13.8) | 29 (23.6) | 1.93 [0.97, 3.84] | 0.06 | 1.03 [0.46, 2.32] | 0.948 | 1.09 [0.65, 1.81] | 0.747 |
| Secondary outcome | |  |  |  |  |  |  |  |  |
|  | mRS 0-2 | 7 (6.4) | 12 (9.8) | 1.58 [0.60, 4.16] | 0.359 | 0.69 [0.22, 2.18] | 0.525 | 0.66 [0.20, 2.18] | 0.498 |
|  | mRS 0-4 | 31 (28.4) | 60 (48.8) | 2.40 [1.39, 4.14] | 0.002 | 1.49 [0.78, 2.85] | 0.223 | 1.52 [0.79, 2.94] | 0.213 |
|  | Ordinal mRS shift (0–6) | 6 [4, 6] | 5 [4, 6] | 2.22 [1.35, 3.57] | 0.001 | 1.23 [0.71, 2.13] | 0.455 | 1.25 [0.71, 2.17] | 0.436 |
| Safety outcome | |  |  |  |  |  |  |  |  |
|  | Mortality | 63 (57.8) | 46 (37.4) | 0.44 [0.26, 0.74] | 0.002 | 0.57 [0.31, 1.05] | 0.072 | 0.54 [0.29, 1.03] | 0.061 |
|  | sICH | 3 (2.8) | 3 (2.4) | 0.88 [0.20, 3.98] | 0.872 | 1.03 [0.23, 4.67] | 0.974 | NA | NA |
| Data are presented as number (percentage) for binary outcomes and median (interquartile range) for the ordinal modified Rankin Scale distribution. The displayed group sizes and outcome summaries correspond to the eligible cohort with observed 90-day outcomes. Odds ratios (ORs), common ORs (cORs), and 95% confidence intervals (CIs) from adjusted models were pooled across the 20 imputed datasets using Rubin’s rules.  ORs compare LAA with CE, with CE as the reference category, within each treatment stratum. For favorable functional outcomes (90-day mRS 0–2, mRS 0–3, and mRS 0–4), an OR greater than 1 indicates higher odds of a favorable outcome with LAA. For mortality and sICH, which were coded as event=1, an OR less than 1 indicates lower odds of the adverse event with LAA. For the ordinal mRS analysis, the cOR was parameterized such that a value greater than 1 indicates a shift toward lower, more favorable mRS scores with LAA.  Missing baseline covariates were handled using multiple imputation by chained equations with 20 imputed datasets, 10 iterations per dataset, and a fixed random seed of 12345. Treatment, etiology, and outcomes were not imputed. Additional details of the imputation procedure are provided in Supplementary Method 3.  In the EVT-treated stratum (n=423; CE, n=277; LAA, n=146), Model 1 adjusted for age, sex, admission glucose, systolic blood pressure, baseline NIHSS score, baseline ASPECTS, intravenous thrombolysis status, occlusion site, and angiographic ASITN/SIR collateral grade. Model 2 included the same covariates and additionally modeled treating center as a random intercept using mixed-effects logistic regression for binary outcomes and mixed-effects proportional-odds regression for ordinal mRS shift.  In the SMT-only stratum (n=232; CE, n=109; LAA, n=123), Model 3 adjusted for age, sex, admission glucose, systolic blood pressure, baseline NIHSS score, baseline ASPECTS, intravenous thrombolysis status, and occlusion site. Model 4 included the same covariates and additionally modeled treating center as a random intercept using mixed-effects logistic regression for binary outcomes and mixed-effects proportional-odds regression for ordinal mRS shift. Angiographic collateral grade was not included because it was unavailable in the SMT-only group.  Because sICH events were sparse, crude and covariate-adjusted estimates for sICH were obtained using Firth penalized logistic regression without a center random effect. sICH was defined according to the Heidelberg Bleeding Classification.  Abbreviations: ASITN/SIR, American Society of Interventional and Therapeutic Neuroradiology/Society of Interventional Radiology collateral grade; ASPECTS, Alberta Stroke Program Early CT Score; CE, cardioembolism; CI, confidence interval; cOR, common odds ratio; EVT, endovascular thrombectomy; IQR, interquartile range; IVT, intravenous thrombolysis; LAA, large-artery atherosclerosis; mRS, modified Rankin Scale; NIHSS, National Institutes of Health Stroke Scale; OR, odds ratio; sICH, symptomatic intracranial hemorrhage; SMT, standard medical therapy. | | | | | | | | | |

## **Supplementary Table 4. Adjusted Associations of Endovascular Thrombectomy With Clinical Outcomes Within Etiologic Subtypes and Etiology-by-Treatment Interactions: Complete-Case and Multiple-Imputation Analyses**

|  | | Complete Case Analysis  EVT vs SMT (Adjusted) | | | | Multiple Imputation (m=20)  EVT vs SMT (Adjusted) | | |
| --- | --- | --- | --- | --- | --- | --- | --- | --- |
|  | | No. (%) | Adjusted effect (95% CI) | P value | P for interaction | Adjusted effect (95% CI) | P value | p for interaction |
| Primary outcome | | |  |  |  |  |  |  |
| mRS 0-3 | |  |  |  | 0.944 |  |  |  |
|  | LAA | 93/262 (35.5) | 2.94 (1.51-5.73) | 0.002 |  | 3.06 (1.58-5.93) | <0.001 | 0.967 |
|  | CE | 103/369 (27.9) | 3.04 (1.46-6.36) | 0.003 |  | 3.00 (1.44-6.25) | 0.003 | 0.967 |
| Secondary outcome | | |  |  |  |  |  |  |
| mRS 0-6 shift analysis | | |  |  | 0.473 |  |  | 0.624 |
|  | LAA | NA | 1.52 (0.94-2.44) | 0.086 |  | 1.57 (0.98-2.52) | 0.06 |  |
|  | CE | NA | 1.92 (1.17-3.15) | 0.010 |  | 1.84 (1.13-3.02) | 0.015 |  |
| mRS 0-2 | |  |  |  | 0.220 |  |  |  |
|  | LAA | 40/262 (15.3) | 2.41 (1.06-5.47) | 0.035 |  | 2.58 (1.15-5.80) | 0.022 | 0.278 |
|  | CE | 69/369 (18.7) | 5.10 (2.02-12.89) | <0.001 |  | 4.99 (1.98-12.57) | <0.001 | 0.278 |
| mRS 0-4 | |  |  |  | 0.158 |  |  |  |
|  | LAA | 138/262 (52.7) | 1.16 (0.63-2.14) | 0.638 |  | 1.22 (0.67-2.24) | 0.519 | 0.273 |
|  | CE | 155/369 (42.0) | 2.09 (1.14-3.84) | 0.017 |  | 1.92 (1.05-3.52) | 0.035 | 0.273 |
| Safety outcomes | | |  |  |  |  |  |  |
| Mortality | |  |  |  | 0.489 |  |  |  |
|  | LAA | 94/262 (35.9) | 0.97 (0.53-1.78) | 0.917 |  | 0.93 (0.50-1.70) | 0.802 | 0.618 |
|  | CE | 181/369 (49.1) | 0.73 (0.42-1.28) | 0.278 |  | 0.76 (0.43-1.33) | 0.337 | 0.618 |
| sICH | |  |  |  | 0.507 |  |  |  |
|  | LAA | 48/262 (18.3) | 7.06 (3.68-13.56) | <0.001 |  | 4.19 (1.26-14.01) | 0.02 | 0.969 |
|  | CE | 72/369 (19.5) | 5.27 (2.94-9.46) | <0.001 |  | 4.33 (1.43-13.14) | 0.01 | 0.969 |
| Values in the “No. (%)” column represent the total number of participants with the specified outcome among all patients within each etiologic subtype, combining the EVT-treated and SMT-only groups. These values are provided to describe the overall outcome frequency within each etiologic subtype and are not treatment-specific event rates.  Adjusted estimates compare endovascular thrombectomy (EVT) plus standard medical therapy with standard medical therapy (SMT) alone within the LAA and CE subgroups. Adjusted odds ratios (ORs) are reported for binary outcomes, and common ORs (cORs) are reported for ordinal mRS shift. For favorable functional outcomes, an OR greater than 1 indicates higher odds of a favorable outcome with EVT. For mortality and symptomatic intracranial hemorrhage, an OR greater than 1 indicates higher odds of the adverse event with EVT. For ordinal mRS shift, a cOR greater than 1 indicates a shift toward lower, more favorable mRS scores with EVT.  Models included treatment, etiology, a treatment-by-etiology interaction term, age, sex, admission glucose, systolic blood pressure, baseline NIHSS score, baseline ASPECTS, intravenous thrombolysis status, and occlusion site. Treating center was modeled as a random intercept for functional outcomes and mortality. Because symptomatic intracranial hemorrhage events were sparse, Firth penalized logistic regression without a center random effect was used for this outcome.  Etiology-specific EVT–outcome associations were derived as simple effects from the fitted interaction models. Interaction P values were obtained using Wald tests.  The complete-case analysis included participants with complete data for all analyzed outcomes and covariates. In the multiple-imputation sensitivity analysis, missing baseline covariates were handled using multiple imputation by chained equations with 20 imputed datasets; estimates and 95% confidence intervals were combined using Rubin’s rules. Treatment, etiology, and outcomes were not imputed.  Abbreviations: ASPECTS, Alberta Stroke Program Early CT Score; CE, cardioembolism; CI, confidence interval; cOR, common odds ratio; EVT, endovascular thrombectomy; IVT, intravenous thrombolysis; LAA, large-artery atherosclerosis; mRS, modified Rankin Scale; NIHSS, National Institutes of Health Stroke Scale; OR, odds ratio; sICH, symptomatic intracranial hemorrhage; SMT, standard medical therapy. | | | | | | | | |

## **Supplementary Table 5. Assessment of the Proportional-Odds Assumption**

| Term | P value | Interpretation |
| --- | --- | --- |
| Omnibus | 0.001† | Evidence of global nonproportionality |
| Individual predictors |  |  |
| Treatment (EVT vs SMT) | 0.023† | Evidence of nonproportionality |
| Age | 0.010† | Evidence of nonproportionality |
| Baseline NIHSS | 0.119 | No evidence of violation |
| Etiology (LAA vs CE) | 0.672 | No evidence of violation |
| Sex (male) | 0.664 | No evidence of violation |
| ASPECTS | 0.570 | No evidence of violation |
| Glucose | 0.757 | No evidence of violation |
| IVT | 0.683 | No evidence of violation |
| Occlusion site M1 | 0.610 | No evidence of violation |
| Occlusion site M2 | 0.986 | No evidence of violation |
| Interaction term: |  |  |
| Treatment × Etiology | 0.399 | No evidence of violation |
| † P value <0.05 indicates evidence that the corresponding coefficient varies across mRS thresholds. The omnibus test evaluates the proportional-odds assumption for the diagnostic model as a whole. The diagnostic was based on a standard proportional-odds model without a treating-center random effect. No evidence of nonproportionality was detected for the treatment-by-etiology interaction term (P=0.399). The corresponding overall interaction estimate is reported in Supplementary Table 4, and cutpoint-specific sensitivity analyses are presented in Supplementary Figure 3. | | |

## **Supplementary Table 6. Ten-Fold Out-of-Fold Performance of the Etiology-Free Pretreatment Prognostic Score for Favorable 90-Day Functional Outcome (mRS 0–3) in the EVT Cohort**

| **Metric** | **Estimate** | **95% CI** | **Notes** |
| --- | --- | --- | --- |
| OOF AUC | 0.794 | [0.749, 0.833] | Subject-level bootstrap (B=1000) |
| CV-AUC (mean ± SD) | 0.796 ± 0.083 | — | Mean±SD of per-fold AUC across 10 folds |
| Calibration slope | 1.94 | [1.57, 2.44] | Slope from logistic recalibration of the OOF predictions |
| Calibration intercept | 0.41 | [0.12, 0.77] | Intercept from logistic recalibration of the OOF predictions |
| N (evaluated) | 423 | — | Number of individuals with OOF predictions |
| Out-of-fold (OOF) predicted probabilities were generated using 10-fold cross-validation in the EVT cohort from a prespecified model containing selected pretreatment clinical and imaging variables, with TOAST etiology excluded by design. The OOF area under the receiver operating characteristic curve (AUC) was calculated using predicted probabilities from all evaluated participants. The cross-validated AUC (CV-AUC) represents the mean and standard deviation of the fold-specific AUC estimates.  Confidence intervals for the OOF AUC and calibration parameters were obtained using subject-level bootstrap resampling with 1,000 replicates applied to the fixed OOF predicted probabilities. This complementary analysis was used to characterize prognostic separation and overlap between LAA and CE based on measured pretreatment features.  Abbreviations: AUC, area under the receiver operating characteristic curve; CI, confidence interval; CV, cross-validation; EVT, endovascular thrombectomy; LAA, large-artery atherosclerosis; CE, cardioembolism; mRS, modified Rankin Scale; OOF, out-of-fold; SD, standard deviation; TOAST, Trial of ORG 10172 in Acute Stroke Treatment. | | | |

**Supplementary Table 7-0. Domain-Level Summary of the Crude-to-Adjusted Change in the LAA–CE Association With 90-Day mRS 0–3 Among EVT-Treated Patients**

| Covariate domain | Signed Shapley contribution to overall attenuation, percentage points | OR for LAA vs CE from the domain-omitted model | Change in absolute log(OR) vs the full model | Likelihood-ratio loss, Δ(−2LL) |
| --- | --- | --- | --- | --- |
| Imaging features (ASPECTS, ASITN/SIR collateral grade, and occlusion site) | 50.4 | 1.321 | 0.259 | 26.273 |
| Demographics (age and sex) | 37.0 | 1.235 | 0.192 | 30.328 |
| Stroke severity (baseline NIHSS) | 26.0 | 1.150 | 0.120 | 24.505 |
| Metabolic (glucose and SBP) | -6.9 | 0.883 | 0.106 | 11.810 |
| Intravenous thrombolysis | -0.6 | 0.974 | 0.007 | 1.544 |
| Treating center (fixed-effect indicators) | -9.6 | 0.946 | 0.036 | 45.769 |
| Within the decomposition model, the crude OR for LAA versus CE was 1.659 and the fully adjusted OR was 0.981, corresponding to a 96.2% reduction in the absolute log-odds magnitude of the crude association.  Shapley/Lindeman–Merenda–Gold contributions were estimated by averaging each domain’s marginal contribution across 2,000 randomly sampled domain-entry orders. Contributions are reported as signed percentage-point contributions to the overall attenuation. Positive values indicate that a domain moved the LAA–CE association toward the null on average, whereas negative values indicate movement away from the null and may reflect correlations among domains or odds-ratio noncollapsibility. Signed contributions sum to the overall attenuation, with minor differences due to rounding.  Domain-omitted models were obtained by refitting the fully adjusted decomposition model after excluding the specified domain. The change in absolute log(OR) was calculated relative to the fully adjusted model. The likelihood-ratio loss compares the fully adjusted model with the corresponding domain-omitted model. Degrees of freedom and P values are reported in Supplementary Table 7-3.  Abbreviations: ASITN/SIR, American Society of Interventional and Therapeutic Neuroradiology/Society of Interventional Radiology collateral grade; ASPECTS, Alberta Stroke Program Early CT Score; CE, cardioembolism; EVT, endovascular thrombectomy; IVT, intravenous thrombolysis; LAA, large-artery atherosclerosis; mRS, modified Rankin Scale; NIHSS, National Institutes of Health Stroke Scale; OR, odds ratio; SBP, systolic blood pressure. | | | | |

### **Supplementary Table 7-1. Sequential Change in the LAA–CE Association With 90-Day mRS 0–3 Among EVT-Treated Patients**

| **Step** | **OR** | **95% CI** | **P** | **Δ (pp)** | **Cumulative attenuation (%)** |
| --- | --- | --- | --- | --- | --- |
| Crude | 1.659 | 1.05–2.62 | 0.016 | — | 0.0 |
| + Demographics | 1.332 | 0.856–2.07 | 0.203 | +43.3 | 43.3 |
| + Stroke severity | 1.120 | 0.700–1.79 | 0.635 | +34.2 | 77.5 |
| + Imaging features | 0.822 | 0.492–1.37 | 0.453 | −16.3 | 61.2 |
| + Metabolic | 0.933 | 0.657–1.32 | 0.794 | +25.0 | 86.2 |
| + Intravenous thrombolysis | 0.946 | 0.559–1.66 | 0.837 | +2.9 | 89.1 |
| + Treating center | 0.981 | 0.540–1.78 | 0.950 | +7.2 | 96.2 |
| The analysis included EVT-treated patients and compared LAA with CE, with CE as the reference category. Logistic regression models were fitted beginning with the crude model containing etiology alone, followed by sequential addition of demographics, stroke severity, imaging features, metabolic/hemodynamic measures, intravenous thrombolysis, and treating center. Treating center was represented by fixed indicator variables in the final step.  Cumulative attenuation represents the percentage reduction in the absolute log-odds magnitude of the crude LAA–CE association after addition of each covariate domain. The incremental change (Δ, percentage points) represents the difference in cumulative attenuation from the preceding model. Positive values indicate movement of the LAA–CE association toward the null, whereas negative values indicate movement away from the null. Because domains were entered in a predefined sequence, these results are order-dependent; order-averaged domain contributions are reported in Supplementary Table 7-2.  ORs, 95% confidence intervals, and P values are Wald-based.  Abbreviations: ASPECTS, Alberta Stroke Program Early CT Score; CE, cardioembolism; EVT, endovascular thrombectomy; IVT, intravenous thrombolysis; LAA, large-artery atherosclerosis; mRS, modified Rankin Scale; NIHSS, National Institutes of Health Stroke Scale; OR, odds ratio; pp, percentage points; SBP, systolic blood pressure. | | | | | |

### **Supplementary Table 7-2. Order-Averaged Shapley/LMG Domain Contributions to the Crude-to-Adjusted Change in the LAA–CE Association**

| **Covariate domain** | **Signed contribution to overall attenuation, percentage points** |
| --- | --- |
| Imaging features | 50.43 |
| Demographics | 36.97 |
| Stroke severity | 25.98 |
| Intravenous thrombolysis | −0.64 |
| Metabolic | −6.93 |
| Treating center | −9.57 |
| Shapley/Lindeman–Merenda–Gold contributions were estimated as average marginal changes in the absolute log-odds magnitude of the LAA–CE association across 2,000 randomly sampled domain-entry orders. Positive values indicate an average contribution toward attenuation of the association, whereas negative values indicate average movement away from the null and may reflect correlations among domains or odds-ratio noncollapsibility. Signed contributions sum to the overall crude-to-adjusted attenuation reported in Supplementary Table 7-1, with minor differences attributable to Monte Carlo variation and rounding.  Abbreviations: LAA, large-artery atherosclerosis; CE, cardioembolism; LMG, Lindeman–Merenda–Gold. | |

### **Supplementary Table 7-3. Drop-One-Domain Likelihood-Ratio Comparisons and Relative Contributions to Model Fit**

| **Covariate domain** | \|  \| \| --- \|   **Likelihood-ratio loss, Δ(−2LL)** | **df** | **P value** | **Relative likelihood contribution** |
| --- | --- | --- | --- | --- | --- |
| Demographics | 30.328 | 2 | 2.60×10⁻7 | 0.1681 |
| Stroke severity | 24.505 | 1 | 7.41×10⁻7 | 0.1358 |
| Imaging features | 26.273 | 5 | 8.36×10⁻6 | 0.1456 |
| Metabolic | 11.810 | 1 | 5.89×10⁻4 | 0.0654 |
| Intravenous thrombolysis | 1.544 | 1 | 0.214 | 0.0086 |
| Treating center | 45.769 | 30 | 0.0327 | 0.2536 |
| Likelihood-ratio tests compared the fully adjusted model with a model omitting the specified covariate domain while retaining all other terms. Degrees of freedom reflect the difference in the number of estimated parameters and therefore account for multilevel categorical variables.  The relative likelihood contribution represents the likelihood-ratio loss after omission of the specified domain as a proportion of the total likelihood-ratio improvement from the crude model to the fully adjusted model. This measure characterizes the contribution of each domain to overall model fit and is distinct from its Shapley contribution to attenuation of the LAA–CE association. Because information may overlap across domains, these values should not be interpreted as mutually exclusive proportions and are not expected to sum to 1.  Abbreviations: df, degrees of freedom; LL, log likelihood. | | | | |

### **Supplementary Table 7-4. Leave-One-Domain-Out Changes in the LAA–CE Association Relative to the Fully Adjusted Model**

| **Omitted covariate domain** | \|  \| \| --- \|   **OR for LAA vs CE (95% CI)** | **Change in absolute log(OR) vs the full model** | **Direction after domain omission** |
| --- | --- | --- | --- | --- |
| Full model | 0.981 (0.540–1.781) | 0 | — |
| Demographics | 1.235 (0.711–2.144) | 0.192 | Away from the null |
| Stroke severity | 1.150 (0.647–2.043) | 0.120 | Away from the null |
| Imaging features | 1.321 (0.647–2.043) | 0.259 | Away from the null |
| Metabolic/hemodynamic measures | 0.883 (0.490–1.592) | 0.106 | Away from the null |
| Intravenous thrombolysis | 0.974 (0.537–1.769) | 0.007 | Away from the null |
| Treating center | 0.946 (0.559–1.603) | 0.036 | Away from the null |
| Each domain-omitted model was obtained by refitting the fully adjusted model after excluding the specified covariate domain while retaining all other terms. The change in absolute log(OR) was calculated relative to the fully adjusted model. A positive value indicates that omission of the domain moved the LAA–CE association farther from the null, consistent with an attenuating contribution of that domain in the fully adjusted model. A negative value would indicate that omission of the domain moved the association toward the null.  Abbreviations: CE, cardioembolism; CI, confidence interval; LAA, large-artery atherosclerosis; OR, odds ratio. | | | |

### **Supplementary Table 7-5. Order-Averaged Shapley/LMG Contributions After Separating the Imaging Domain Into Collateral Grade, ASPECTS, and Occlusion Site**

| **Block** | \|  \| \| --- \|   **Signed contribution to overall attenuation, percentage points** |
| --- | --- | --- |
| Demographics | 39.95 |
| ASITN/SIR collateral grade | 36.49 |
| Stroke severity | 26.67 |
| ASPECTS | 6.28 |
| Occlusion site | 5.49 |
| Intravenous thrombolysis | −0.91 |
| Metabolic/hemodynamic measures | −8.02 |
| Treating center | −9.71 |
| The combined imaging domain was separated into angiographic ASITN/SIR collateral grade, baseline ASPECTS, and occlusion site, and the Shapley/Lindeman–Merenda–Gold decomposition was repeated. Contributions were estimated as average marginal changes in the absolute log-odds magnitude of the LAA–CE association across 2,000 randomly sampled domain-entry orders and are reported as signed percentage-point contributions to the overall attenuation.  Among the imaging components, collateral grade had the largest positive contribution, whereas ASPECTS and occlusion site made smaller positive contributions. Negative values indicate average movement of the LAA–CE association away from the null and may reflect correlations among domains or odds-ratio noncollapsibility. Signed contributions sum to the overall crude-to-adjusted attenuation, with minor differences due to Monte Carlo variation and rounding.  Abbreviations: ASITN/SIR, American Society of Interventional and Therapeutic Neuroradiology/Society of Interventional Radiology collateral grade; ASPECTS, Alberta Stroke Program Early CT Score; CE, cardioembolism; LAA, large-artery atherosclerosis; LMG, Lindeman–Merenda–Gold. | |

**Supplementary Table 7-6. Marginal Standardized Risks, Risk Difference, and Risk Ratio for 90-Day mRS 0–3 by Etiology**

| **Metric** | **Estimate** | **95% CI** |
| --- | --- | --- |
| Standardized risk, CE | 0.375 | 0.3189–0.4310 |
| Standardized risk, LAA | 0.372 | 0.2956–0.4586 |
| Risk difference, LAA minus CE | −0.0028 | −0.0968–0.0912 |
| Risk ratio, LAA vs CE | 0.9926 | 0.7615–1.2480 |
| As a sensitivity analysis on marginal risk scales, standardized outcome probabilities were estimated from the fully adjusted fixed-effects logistic regression model, including treating-center indicator variables, in the complete-case EVT cohort. For each participant, model-based predicted probabilities were generated under LAA and CE coding while all measured covariates were retained at their observed values. Predicted probabilities were then averaged across participants to obtain marginal standardized risks. The risk difference and risk ratio were derived from these standardized risks. Confidence intervals were estimated using nonparametric bootstrap resampling with 1,000 replicates.  Abbreviations: CE, cardioembolism; CI, confidence interval; EVT, endovascular thrombectomy; LAA, large-artery atherosclerosis; mRS, modified Rankin Scale. | | |

# **Supplementary Figure legends**

## **Supplementary Figure 1. Missing Data Pattern in Baseline Covariates**


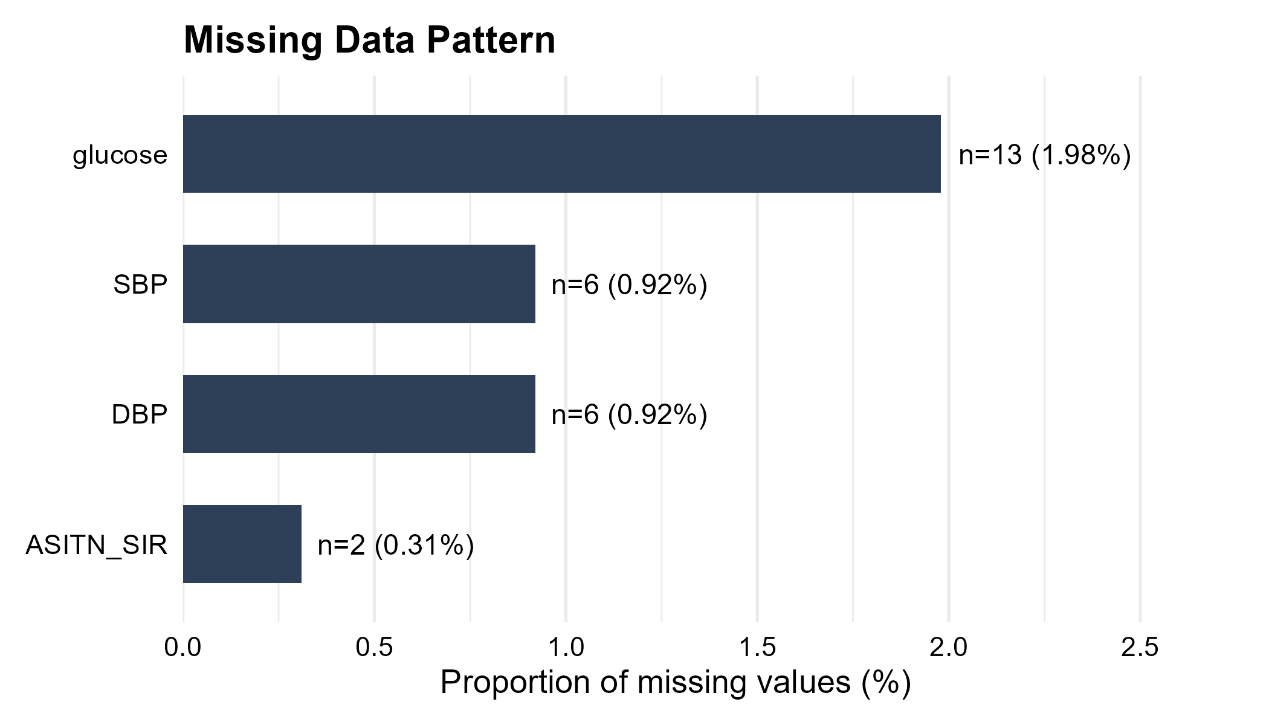
Proportion of missing values (%) for key baseline covariates in the analysis cohort (n=655). Overall missingness was minimal, ranging from 0.31% (ASITN/SIR collateral grade) to 1.98% (admission glucose). Missing baseline covariates were handled using complete-case analysis for primary models; sensitivity analyses using multiple imputation by chained equations (MICE) are reported in supplementary tables.

Abbreviations: ASITN/SIR, American Society of Interventional and Therapeutic Neuroradiology/Society of Interventional Radiology collateral grading; SBP, systolic blood pressure; DBP, diastolic blood pressure.

## **Supplementary Figure 2. Diagnostics for Multiple Imputation of Baseline Covariates**


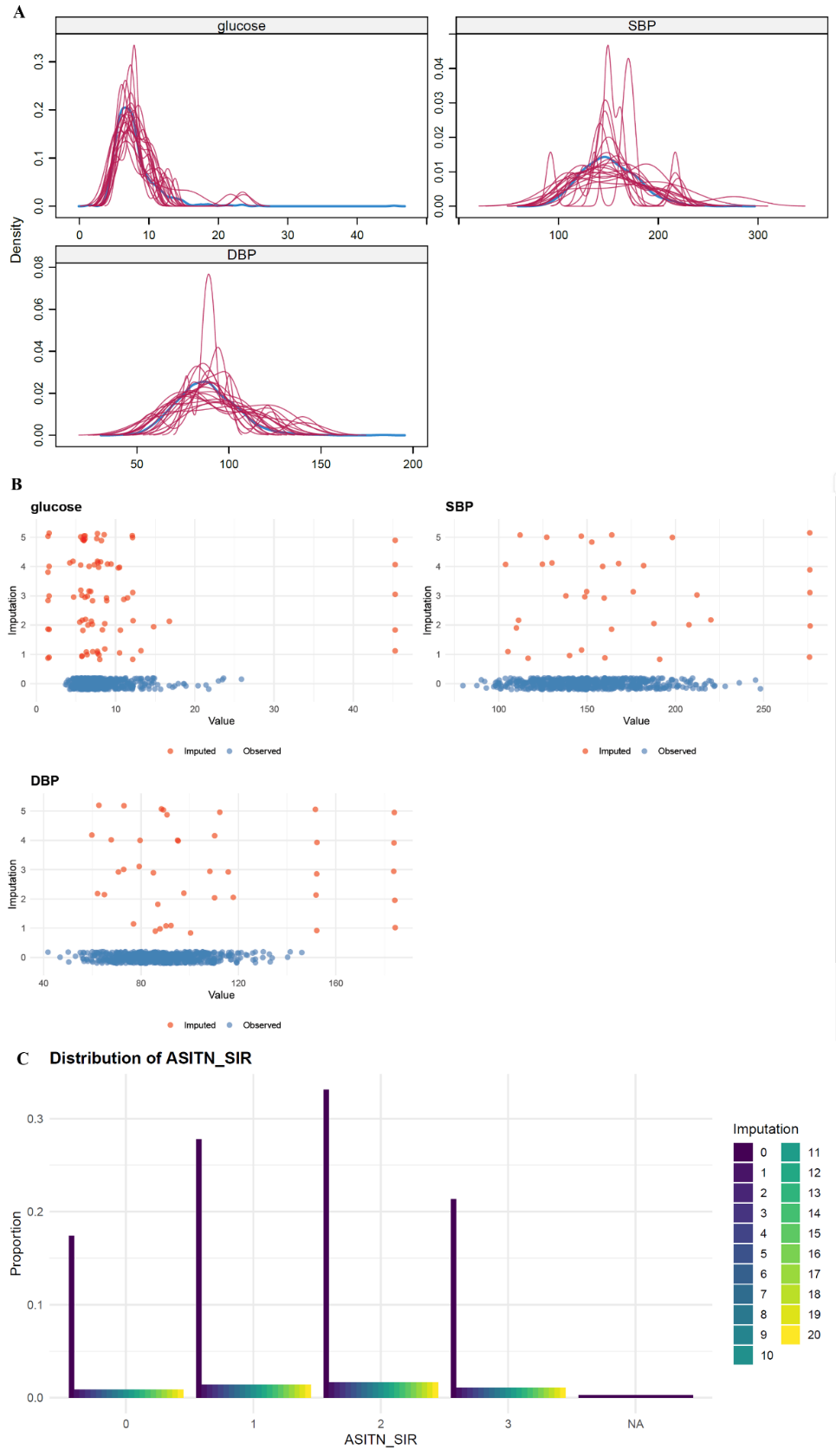


Diagnostic plots for the 20 datasets generated using multiple imputation by chained equations (MICE), with 10 iterations per dataset and a fixed random seed of 12345.

**Panel A:** Kernel density plots comparing observed and imputed values for admission glucose, systolic blood pressure, and diastolic blood pressure. The broadly overlapping distributions indicate that the imputed values were consistent with the ranges and distributions of the observed data.

**Panel B:** Scatter plots of imputation index against covariate values for the same variables. Observed values are shown at index 0, and imputed values are shown across the 20 imputed datasets. Imputed values remained within clinically plausible ranges without evident extreme or impossible values.

**Panel C:** Distribution of ASITN/SIR collateral grade across the 20 imputed datasets. The distributions were stable across imputations. Structural unavailability of angiographic collateral grading in the SMT-only group was retained, whereas missing values among EVT-treated patients were handled within the imputation procedure.

Together, the diagnostic plots supported the plausibility and stability of the imputed baseline covariates. Corresponding multiple-imputation sensitivity analyses are reported in Supplementary Table 3.

Abbreviations: ASITN/SIR, American Society of Interventional and Therapeutic Neuroradiology/Society of Interventional Radiology collateral grade; EVT, endovascular thrombectomy; MICE, multiple imputation by chained equations; SMT, standard medical therapy.

## **Supplementary Figure 3. Adjusted EVT–Outcome Associations Across mRS Thresholds by Stroke Etiology**

**
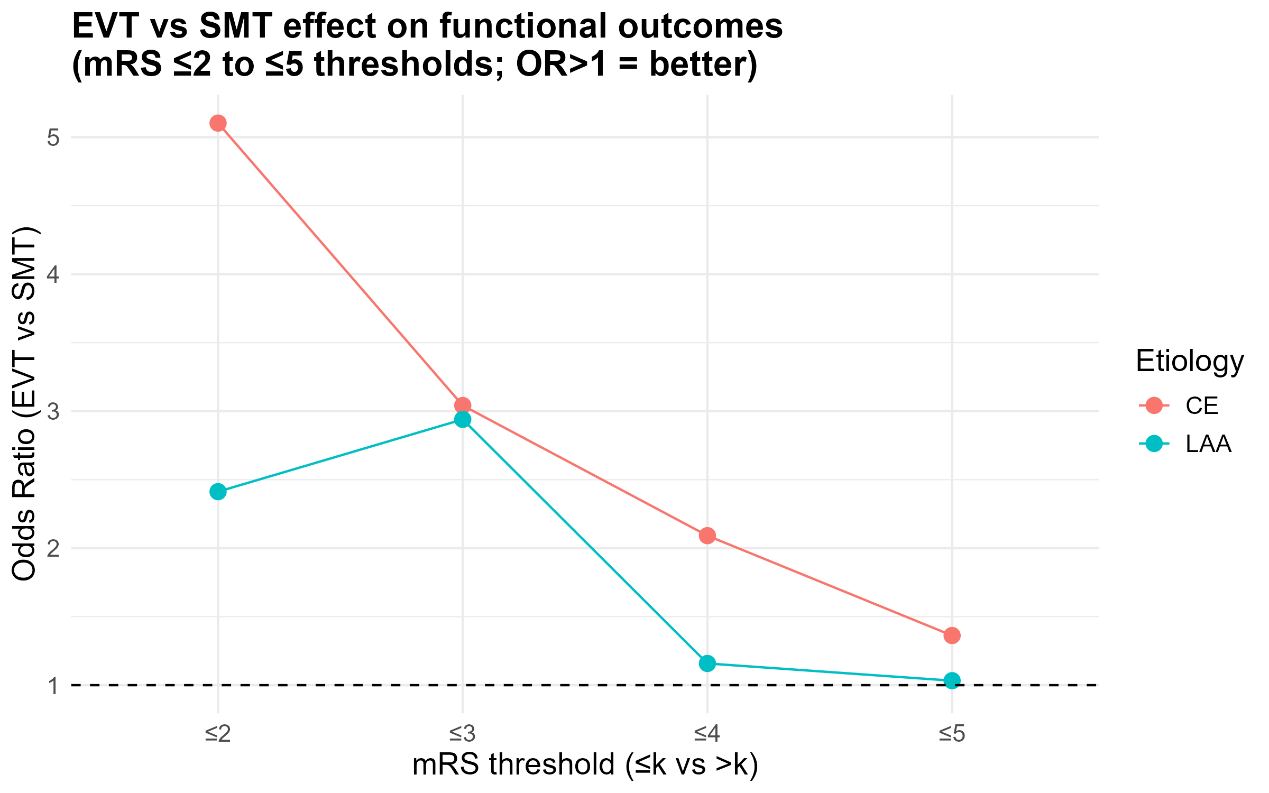
**

Points and horizontal lines show adjusted odds ratios (aORs) with 95% confidence intervals (CIs) for endovascular thrombectomy (EVT) plus standard medical therapy versus standard medical therapy alone within the cardioembolism (CE) and large-artery atherosclerosis (LAA) subgroups. Outcomes were defined at successive 90-day modified Rankin Scale (mRS) thresholds (mRS ≤2, ≤3, ≤4, and ≤5).

Each threshold was analyzed using a mixed-effects logistic regression model containing treatment, etiology, their interaction, age, sex, admission glucose, systolic blood pressure, baseline National Institutes of Health Stroke Scale score, baseline Alberta Stroke Program Early CT Score, intravenous thrombolysis status, and occlusion site, with treating center modeled as a random intercept. Etiology-specific EVT–outcome associations were derived as simple effects from the fitted interaction models. An aOR greater than 1 indicates higher odds of achieving the specified mRS threshold with EVT.

Cutpoint-specific analyses were performed as a sensitivity analysis because the Brant-type diagnostic indicated global nonproportionality in the ordinal mRS model. The adjusted EVT–outcome associations varied in magnitude across thresholds, but no treatment-by-etiology interaction was detected at any threshold (all P for interaction ≥0.158).

Abbreviations: aOR, adjusted odds ratio; ASPECTS, Alberta Stroke Program Early CT Score; CE, cardioembolism; CI, confidence interval; EVT, endovascular thrombectomy; IVT, intravenous thrombolysis; LAA, large-artery atherosclerosis; mRS, modified Rankin Scale; NIHSS, National Institutes of Health Stroke Scale; SMT, standard medical therapy.

**Supplementary Figure 4. Distribution of Out-of-Fold Predicted Probabilities for Favorable 90-Day Functional Outcome by Etiologic Subtype**


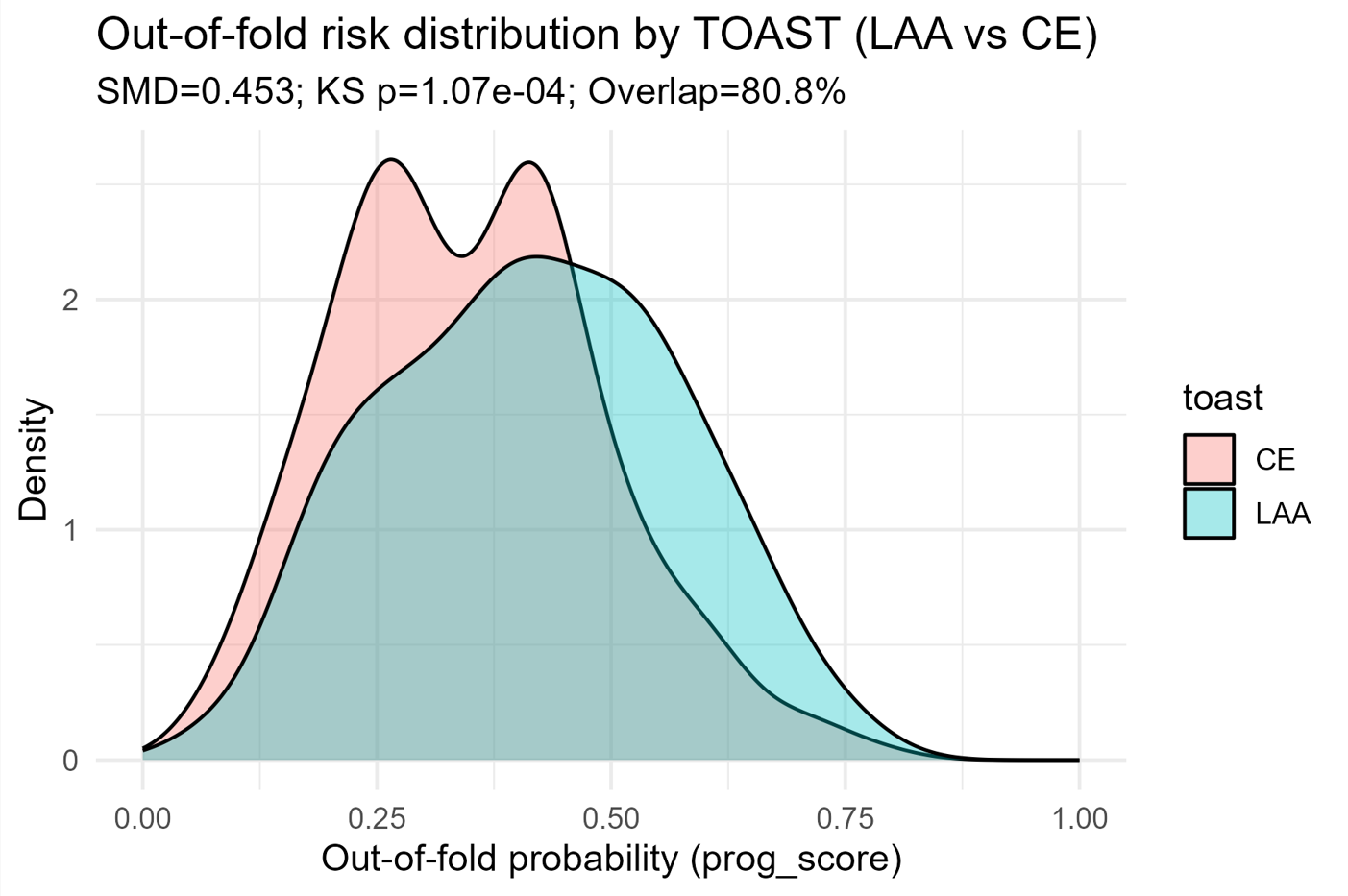


An etiology-free pretreatment prognostic score was generated using 10-fold out-of-fold (OOF) predictions for favorable 90-day functional outcome, defined as a modified Rankin Scale score of 0–3. The regularized logistic regression model included age, sex, admission glucose, baseline National Institutes of Health Stroke Scale score, baseline Alberta Stroke Program Early CT Score, occlusion site, intravenous thrombolysis status, angiographic ASITN/SIR collateral grade, and treating center, with TOAST etiology excluded by design.

Kernel density estimates of the OOF predicted probabilities are shown for large-artery atherosclerosis (LAA) and cardioembolism (CE). The LAA distribution was shifted toward higher predicted probabilities relative to CE (standardized mean difference, 0.453; Kolmogorov–Smirnov P=1.07×10⁻⁴), while the two distributions showed substantial overlap (80.8%). Complementary empirical cumulative distribution function results are shown in Supplementary Figure 5.

Abbreviations: ASITN/SIR, American Society of Interventional and Therapeutic Neuroradiology/Society of Interventional Radiology collateral grade; CE, cardioembolism; LAA, large-artery atherosclerosis; OOF, out-of-fold; TOAST, Trial of ORG 10172 in Acute Stroke Treatment.

## **Supplementary Figure 5. Empirical Cumulative Distribution of Out-of-Fold Predicted Probabilities for Favorable 90-Day Functional Outcome by Etiologic Subtype**


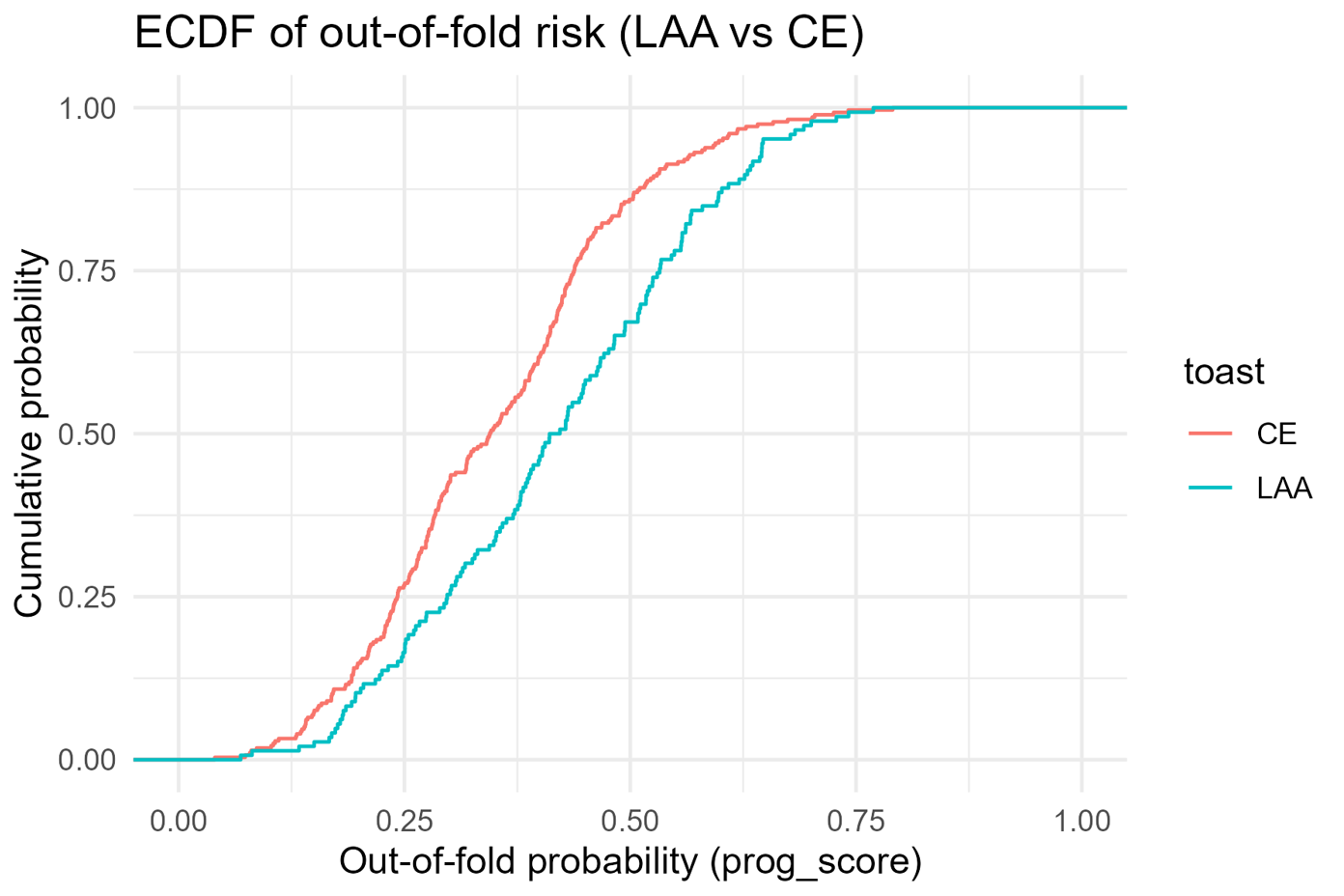


Empirical cumulative distribution functions (ECDFs) are shown for the etiology-free out-of-fold (OOF) predicted probabilities of favorable 90-day functional outcome, defined as a modified Rankin Scale (mRS) score of 0–3, among patients with large-artery atherosclerosis (LAA) and cardioembolism (CE). At each predicted-probability threshold on the x-axis, the y-axis represents the proportion of participants with an OOF predicted probability less than or equal to that threshold.

The CE ECDF was generally higher than the LAA ECDF at the same probability thresholds, indicating that predicted probabilities of favorable outcome were higher on average in LAA. The maximum vertical separation between the curves corresponds to the two-sample Kolmogorov–Smirnov statistic (P=1.07×10⁻⁴). Despite this distributional difference, the predicted-probability distributions overlapped substantially (80.8%). The complementary kernel density display is presented in Supplementary Figure 4, and performance metrics for the etiology-free pretreatment prognostic score are reported in Supplementary Table 6.

Abbreviations: CE, cardioembolism; ECDF, empirical cumulative distribution function; LAA, large-artery atherosclerosis; mRS, modified Rankin Scale; OOF, out-of-fold.

## **Supplementary Figure 6. Sequential Crude-to-Adjusted Change in the LAA–CE Association Among EVT-Treated Patients**


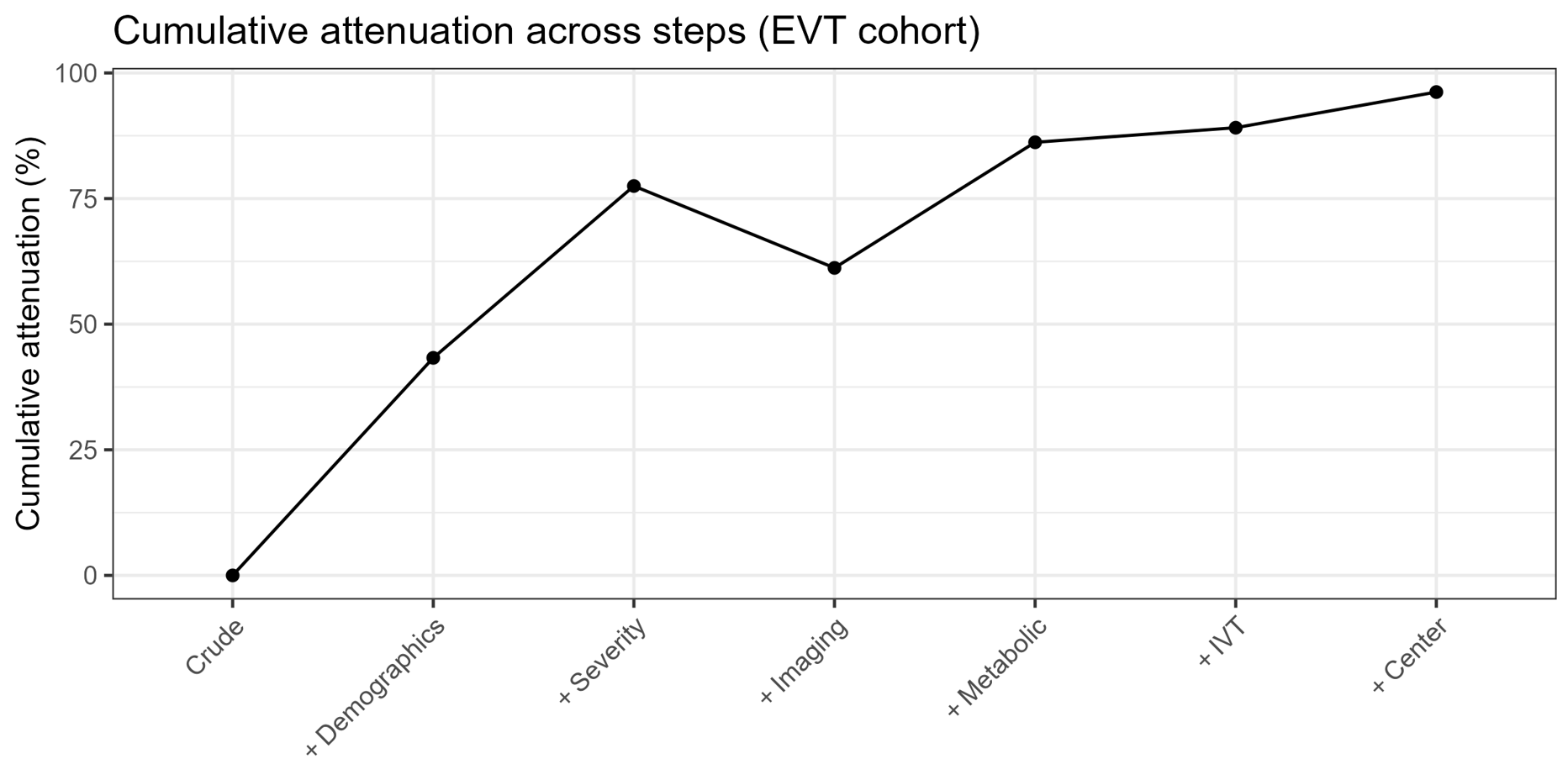


The x-axis shows the predefined sequence of models: crude model, followed by addition of demographics, stroke severity, imaging features, metabolic/hemodynamic measures, intravenous thrombolysis, and treating center. The y-axis shows the cumulative percentage reduction in the absolute log-odds magnitude of the crude LAA–CE association. The cumulative attenuation reached 96.2% after inclusion of all covariate domains. Because domains were added in a predefined sequence, the trajectory is order-dependent; detailed estimates are reported in Supplementary Table 7-1.

Abbreviations: CE, cardioembolism; EVT, endovascular thrombectomy; LAA, large-artery atherosclerosis.

## **Supplementary Figure 7. Order-Averaged Shapley/LMG Contributions of Covariate Domains to the Crude-to-Adjusted Change in the LAA–CE Association**


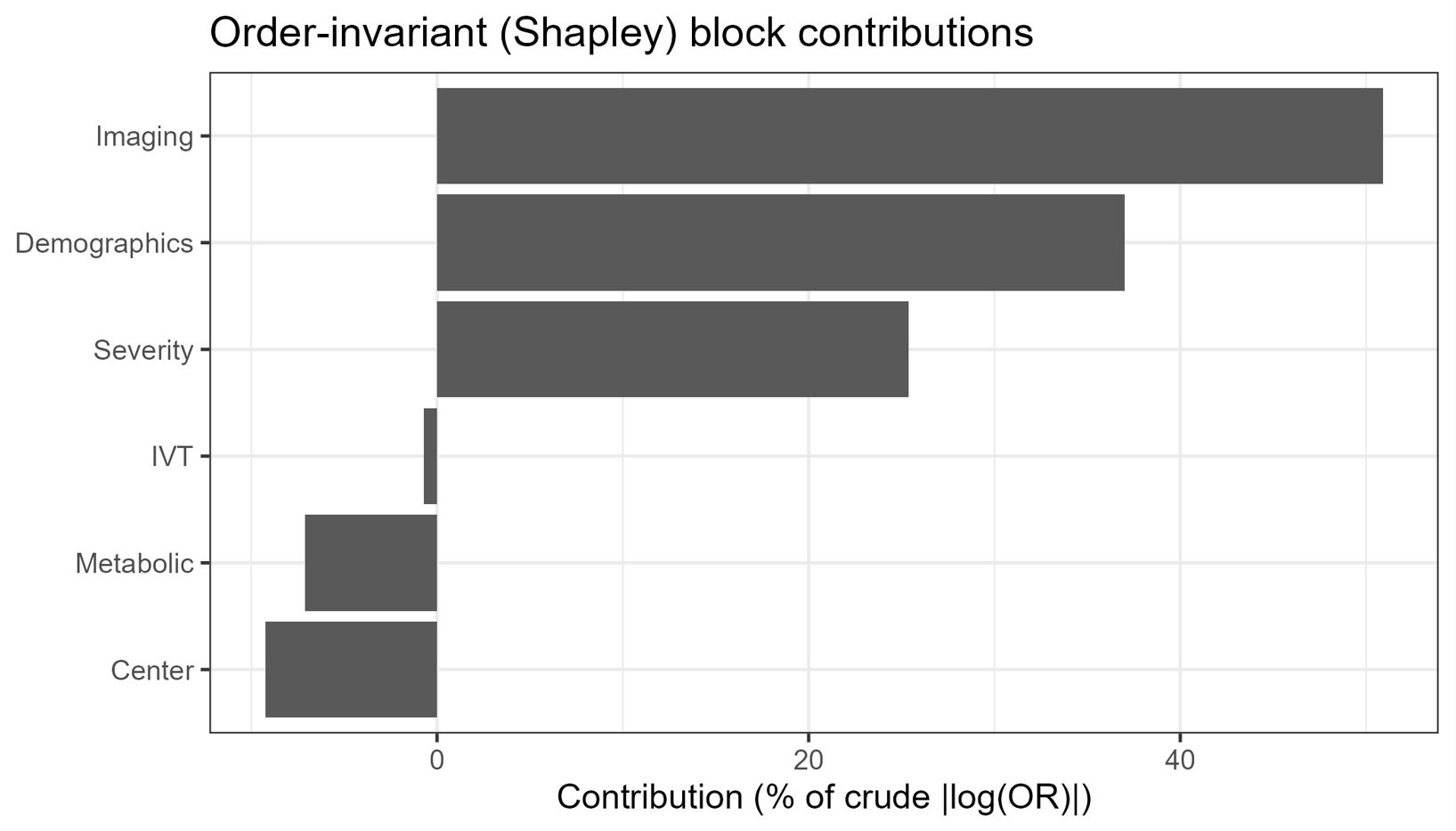
The x-axis shows signed percentage-point contributions to the overall attenuation in the absolute log-odds magnitude of the crude LAA–CE association, and the y-axis shows the covariate domains. Contributions were estimated by averaging each domain’s marginal contribution across 2,000 randomly sampled domain-entry orders. Positive values indicate an average contribution toward attenuation of the LAA–CE association, whereas negative values indicate average movement away from the null. Detailed estimates are reported in Supplementary Table 7-2.

Abbreviations: CE, cardioembolism; LAA, large-artery atherosclerosis; LMG, Lindeman–Merenda–Gold.

## **Supplementary Figure 8. Leave-One-Domain-Out Changes in the LAA–CE Association Relative to the Fully Adjusted Model**


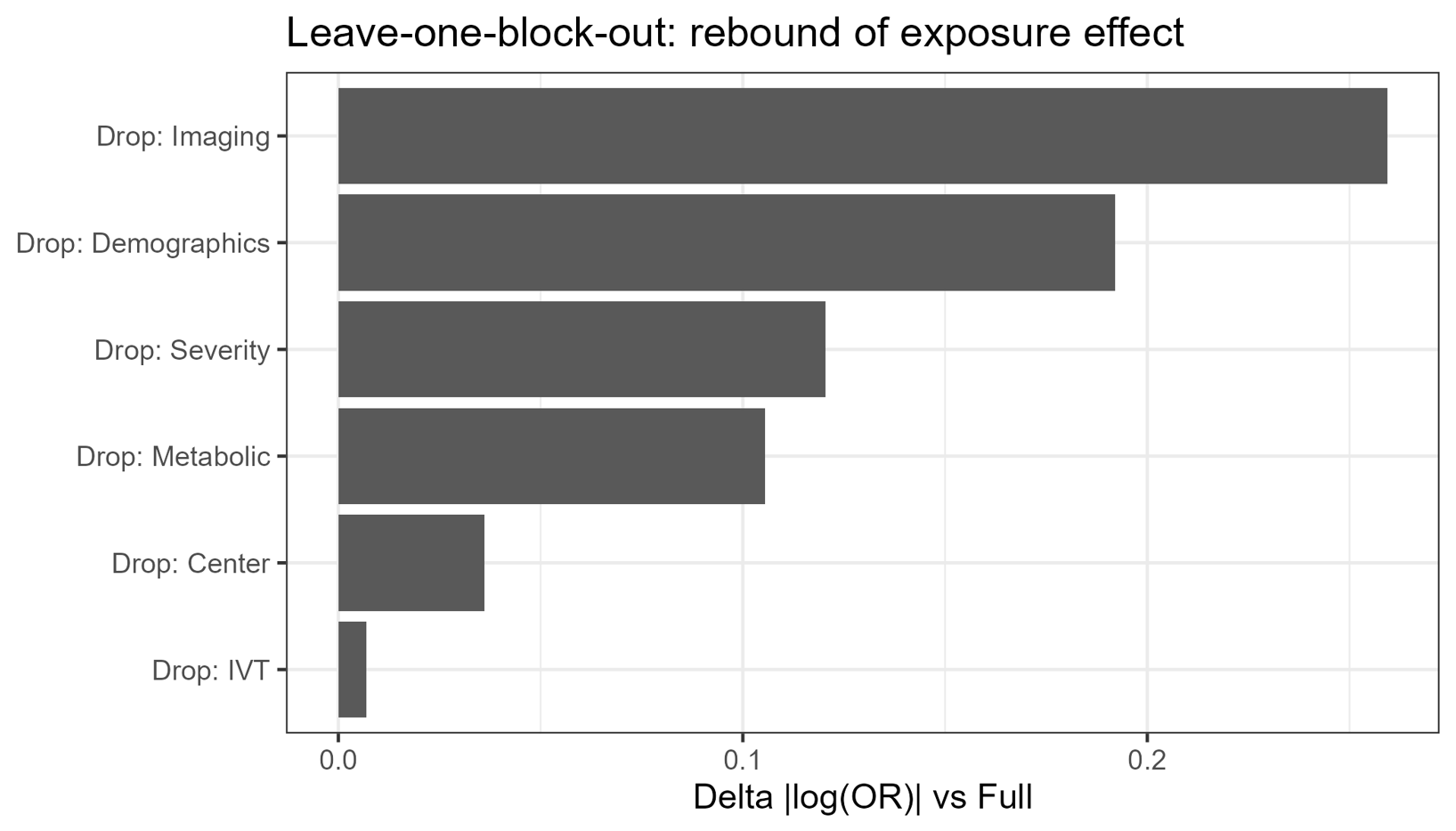
The x-axis shows the change in the absolute log-odds magnitude of the LAA–CE association after omission of each covariate domain relative to the fully adjusted model, and the y-axis shows the omitted domains. Larger positive values indicate that omission of the domain moved the LAA–CE association farther from the null, consistent with a greater attenuating contribution of that domain in the fully adjusted model. Detailed estimates are reported in Supplementary Table 7-4.

Abbreviations: CE, cardioembolism; LAA, large-artery atherosclerosis; OR, odds ratio.

## **Supplementary Figure 9. Order-Averaged Shapley/LMG Contributions After Separating the Imaging Domain**


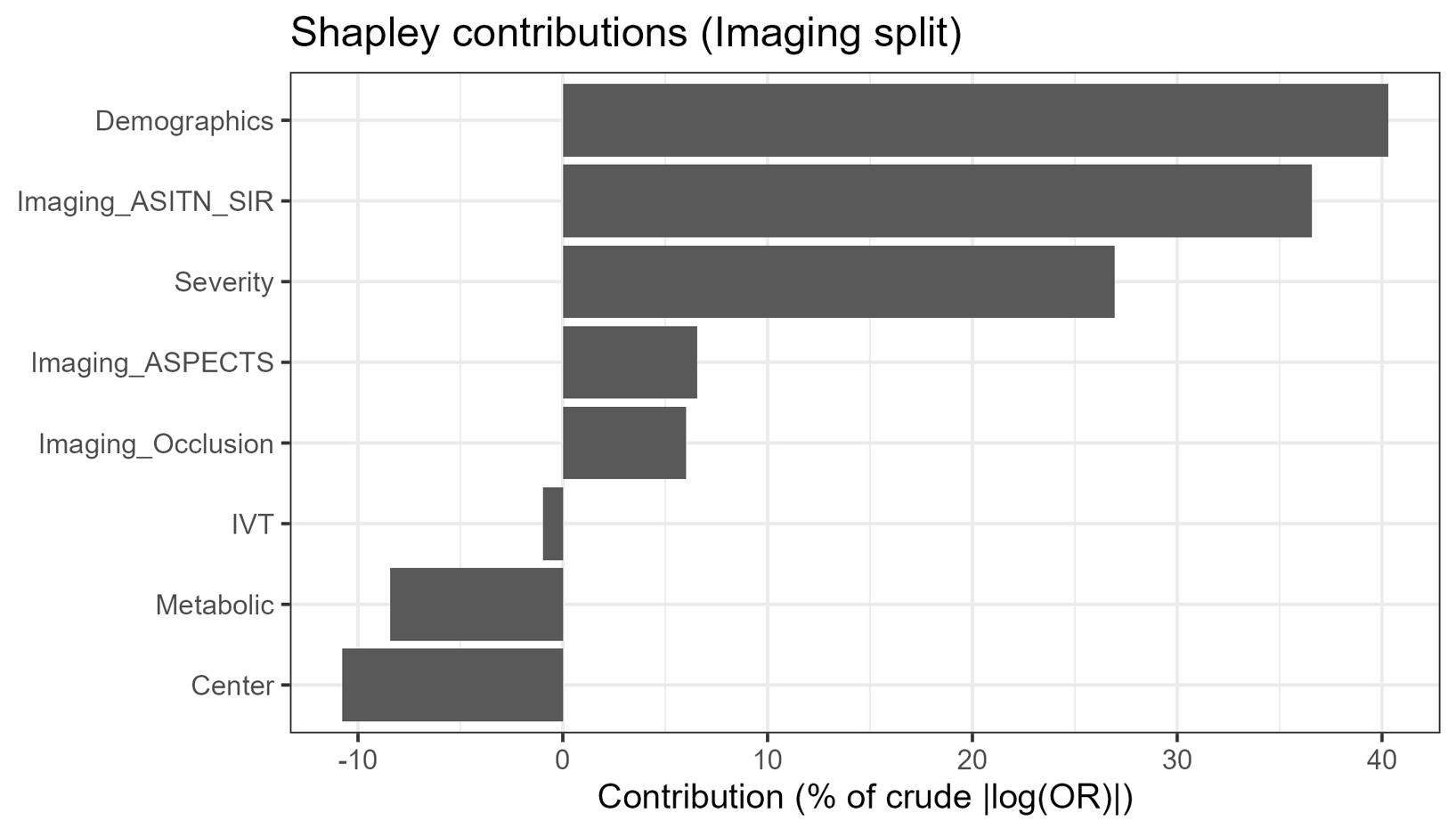


The x-axis shows signed percentage-point contributions to the overall attenuation in the absolute log-odds magnitude of the crude LAA–CE association, and the y-axis shows the covariate domains and individual imaging components. Within the imaging domain, angiographic ASITN/SIR collateral grade had the largest positive contribution, whereas baseline ASPECTS and occlusion site made smaller positive contributions. Detailed estimates are reported in Supplementary Table 7-5.

Abbreviations: ASITN/SIR, American Society of Interventional and Therapeutic Neuroradiology/Society of Interventional Radiology collateral grade; ASPECTS, Alberta Stroke Program Early CT Score; CE, cardioembolism; LAA, large-artery atherosclerosis; LMG, Lindeman–Merenda–Gold.

**References**

[1] Guo C, Li L, Huang J, et al. Endovascular treatment versus standard medical treatment in patients with established large infarct: a cohort study. Int J Surg. 2024. 110(8): 4775-4784.

[2] Adams HP, Bendixen BH, Kappelle LJ, et al. Classification of subtype of acute ischemic stroke. Definitions for use in a multicenter clinical trial. TOAST. Trial of Org 10172 in Acute Stroke Treatment. Stroke. 1993. 24(1): 35-41.

[3] Brott T, Adams HP, Olinger CP, et al. Measurements of acute cerebral infarction: a clinical examination scale. Stroke. 1989. 20(7): 864-70.

[4] Barber PA, Demchuk AM, Zhang J, Buchan AM. Validity and reliability of a quantitative computed tomography score in predicting outcome of hyperacute stroke before thrombolytic therapy. ASPECTS Study Group. Alberta Stroke Programme Early CT Score. Lancet. 2000. 355(9216): 1670-4.

[5] Higashida RT, Furlan AJ, Roberts H, et al. Trial design and reporting standards for intra-arterial cerebral thrombolysis for acute ischemic stroke. Stroke. 2003. 34(8): e109-37.

[6] Zaidat OO, Yoo AJ, Khatri P, et al. Recommendations on angiographic revascularization grading standards for acute ischemic stroke: a consensus statement. Stroke. 2013. 44(9): 2650-63.

[7] van Swieten JC, Koudstaal PJ, Visser MC, Schouten HJ, van Gijn J. Interobserver agreement for the assessment of handicap in stroke patients. Stroke. 1988. 19(5): 604-7.

[8] von Kummer R, Broderick JP, Campbell BC, et al. The Heidelberg Bleeding Classification: Classification of Bleeding Events After Ischemic Stroke and Reperfusion Therapy. Stroke. 2015. 46(10): 2981-6.

[9] White IR, Royston P, Wood AM. Multiple imputation using chained equations: Issues and guidance for practice. Stat Med. 2011. 30(4): 377-99.

[10] van Buuren S, Groothuis-Oudshoorn K. mice: Multivariate Imputation by Chained Equations in R. Journal of Statistical Software. 2011. 45(3): 1 - 67.

[11] Rubin DB. Multiple Imputation for Nonresponse in Surveys. Wiley Series in Probability and Statistics. 1987 .

[12] Heinze G, Schemper M. A solution to the problem of separation in logistic regression. Stat Med. 2002. 21(16): 2409-19.

[13] Brant R. Assessing proportionality in the proportional odds model for ordinal logistic regression. Biometrics. 1990. 46(4): 1171-8.

[14] Friedman J, Hastie T, Tibshirani R. Regularization Paths for Generalized Linear Models via Coordinate Descent. J Stat Softw. 2010. 33(1): 1-22.

[15] Pastore M, Calcagnì A. Measuring Distribution Similarities Between Samples: A Distribution-Free Overlapping Index. Front Psychol. 2019. 10: 1089.

[16] Williamson BD, Feng J. Efficient nonparametric statistical inference on population feature importance using Shapley values. Proc Mach Learn Res. 2020. 119: 10282-10291.

[17] Groemping U. Relative Importance for Linear Regression in R: The Package relaimpo. Journal of Statistical Software. 2006. 17(1): 1 - 27.

[18] Muller CJ, MacLehose RF. Estimating predicted probabilities from logistic regression: different methods correspond to different target populations. Int J Epidemiol. 2014. 43(3): 962-70.

[19] Localio AR, Margolis DJ, Berlin JA. Relative risks and confidence intervals were easily computed indirectly from multivariable logistic regression. J Clin Epidemiol. 2007. 60(9): 874-82.
